# Real-World Safety of Anti-EGFR Antibodies: 20-Year Pharmacovigilance of Cetuximab and Panitumumab

**DOI:** 10.7150/ijms.122194

**Published:** 2025-09-29

**Authors:** Liang-Yan Jin, Guo-Quan Chen, Jun Xie, Ping Chen, Hua-Yu Sun, Hong-Liang Zheng, Liu-Cheng Li, Kai-Li Mao

**Affiliations:** 1 Hangzhou Xixi Hospital, Hangzhou Sixth People's Hospital, Hangzhou Xixi Hospital Affiliated to Zhejiang Chinese Medical University, Hangzhou 310023, Zhejiang, China.; 2 Department of Pharmacy, Affiliated Jinhua Hospital, Zhejiang University School of Medicine, Jinhua 321000, China.; 3 The Quzhou Affiliated Hospital of Wenzhou Medical University, Quzhou People's Hospital, Quzhou 324000, China.; 4 Department of Pharmacy, Sir Run Run Shaw Hospital, Zhejiang University School of Medicine, Hangzhou 310016, China.; Liang-Yan Jin, Guo-Quan Chen, and Kai-Li Mao contributed equally to this work.

**Keywords:** colorectal cancer, cetuximab, panitumumab, adverse events

## Abstract

**Background:** Colorectal cancer remains a major contributor to global cancer-related mortality. Although EGFR-targeted monoclonal antibodies (cetuximab, panitumumab) have demonstrated efficacy in improving clinical outcomes, their associated adverse events underscore the need for enhanced prevention and management strategies. This study analyses real-world post-marketing data to enhance patient safety.

**Methods:** The FDA Adverse Event Reporting System database served as the data source for this study, which centered on AEs related to cetuximab and panitumumab from 2004 to 2024. Standardized MedDRA queries are performed to fully search for AEs at the preferred term (PT) level. The analysis applied the reporting odds ratio (ROR) to identify AEs signals. The reliability of the findings was reinforced by employing multivariate logistic regression to handle confounders.

**Results:** The analysis included 19,131 cetuximab primary suspect cases with 50,338 adverse events (AEs) and 9,448 panitumumab primary suspect cases with 30,061 AEs. The incidence of AEs for cetuximab in 24 organ systems and for panitumumab in 23 organ systems. Cetuximab had higher AE frequency in skin, vascular, and respiratory disorders, with the majority of skin-related AEs occurring within 15 days. Panitumumab showed stronger links to hypomagnesemia and hepatobiliary disorders, and and neurotoxicity, with skin AEs appearing around 28 days. Notably, panitumumab exhibited a higher risk of AEs compared to cetuximab and revealed a novel signal for hepatobiliary disorders.

**Conclusion:** The study demonstrates substantial variations in the safety of cetuximab and panitumumab, emphasizing the need for tailored monitoring, prudent interchangeability, and management strategies in clinical practice. Future research should explore the underlying mechanisms and targeted interventions to improve patients' outcomes and quality of life.

## Introduction

On a global scale, malignancies are poised to assume the role of the primary agent responsible for a major burden of life loss before their time, with a notable likelihood of overtaking ailments of the cardiovascular system as the foremost contributor to such premature mortality trends^1^-^3^. Colorectal cancer (CRC) ranks as one of the most intense, multifaceted, and lethal forms of cancer^4^, ^5^. According to 2020 global cancer statistics, CRC is the second deadliest type of cancer and is diagnosed in third place most frequently among all cancers globally^6^. Among individuals afflicted with CRC, the hepatic and pulmonary systems emerge as the predominant sites of metastatic dissemination^7^, ^8^. Notably, roughly 25% of patients display signs of metastatic disease upon initial diagnosis, whereas as many as 60% may ultimately develop distant metastases over a span of five years^7^, ^8^. The proportion of patients who achieve a 5-year survival milestone often falling below the 20% threshold with regard to metastatic CRC (mCRC)^9^.

In the last two decades, the prognosis for mCRC patients has gotten better^10^. This progress is due to more surgical removal of metastases and the emergence of new medications^11^. In mCRC, The excessive presence of EGFR and its ligands correlates with a worse prognosis and more aggressive disease^12^. In the early 1980s, targeting the EGFR pathway with monoclonal antibodies (mAbs) became a viable treatment option^13^. Combining targeted therapy alongside irinotecan- and oxaliplatin-based therapy for mCRC patients can increase their median overall survival to over two years, making more patients with chemotherapy-refractory conditions to qualify for third-line treatment^14^, ^15^. Patients experiencing resistance to chemotherapy in the context of mCRC can find clinical advantages through the administration of anti-EGFR mAbs. Cetuximab, a chimeric immunoglobulin G1 antibody, and panitumumab, a fully human immunoglobulin G2 antibody, are the specific agents that provide these benefits. Cetuximab and panitumumab have received approval from North American and European regulatory bodies for treating advanced CRC^16^-^19^. Notwithstanding their significant effectiveness in managing mCRC, the associated adverse events (AEs) warrant vigilant attention. mCRC patients who are administered regimens with anti-EGFR mAbs have an approximately 1.5-fold increased risk of experiencing venous or pulmonary embolism compared to those not treated with anti-EGFR mAbs^20^. Patients with mCRC who received anti-EGFR monoclonal antibodies therapy reported experiencing skin toxicity of grade 3 to 4 (panitumumab 13% *vs*. cetuximab 10%), infusion reactions (panitumumab < 0.5% *vs*. cetuximab 2%), and hypomagnesaemia (panitumumab 7% *vs*. cetuximab 3%)^21^. Additionally, the study also noted one treatment-associated fatal adverse event: a lung infection in a patient who received cetuximab.

Emerging research on the AEs linked to the widespread usage of anti-EGFR mAbs has been reported^22^, ^23^. Nevertheless, the investigation through clinical trials has predominantly involved small sample sizes, randomized controlled trials with stringent inclusion criteria, or has been restricted to specific organs. It has inadvertently masked the detection of certain potentially hazardous safety issues. Therefore, conducting a methodical examination of AEs associated with anti-EGFR mAbs in a large-scale patient population is crucial for thoroughly assessing the safety characteristics of anti-EGFR mAbs. This research was to apply the standardized real-world data provided by the FDA Adverse Event Reporting System (FAERS) to assess the safety profiles associated with cetuximab and panitumumab.

## Methods

### Data sources

The post-marketing surveillance program for pharmaceuticals and therapeutic biological products, operated by the FDA, relies on voluntary reports that are integrated into the FAERS database. These reports come from diverse sources such as patients, pharmacists, and healthcare professionals. It comprises patient demographic data, medication usage details, adverse reaction specifics, reporting source information, therapy duration, drug indications, and patient outcome data. Seven unique types of data documents were discovered in the FAERS data files: DRUG (drug details), OUTC (patient outcomes), RPSR (reporting sources), THER (therapy start and end dates for drugs), INDI (drug administration indications), and DEMO (demographic and administrative data). In the FAERS database structure, these documents are interconnected using unique identifiers such as PRIMARYID.

The current study employed information sourced from the FAERS database, which is publicly available and contains anonymized data. This database is specifically designed to aid the drug safety and is available for research purposes. Given the nature of the data, this research did not demand Institutional Review Board approval, nor did it require patient consent or approval.

### Data Extraction

**Figure 1:**
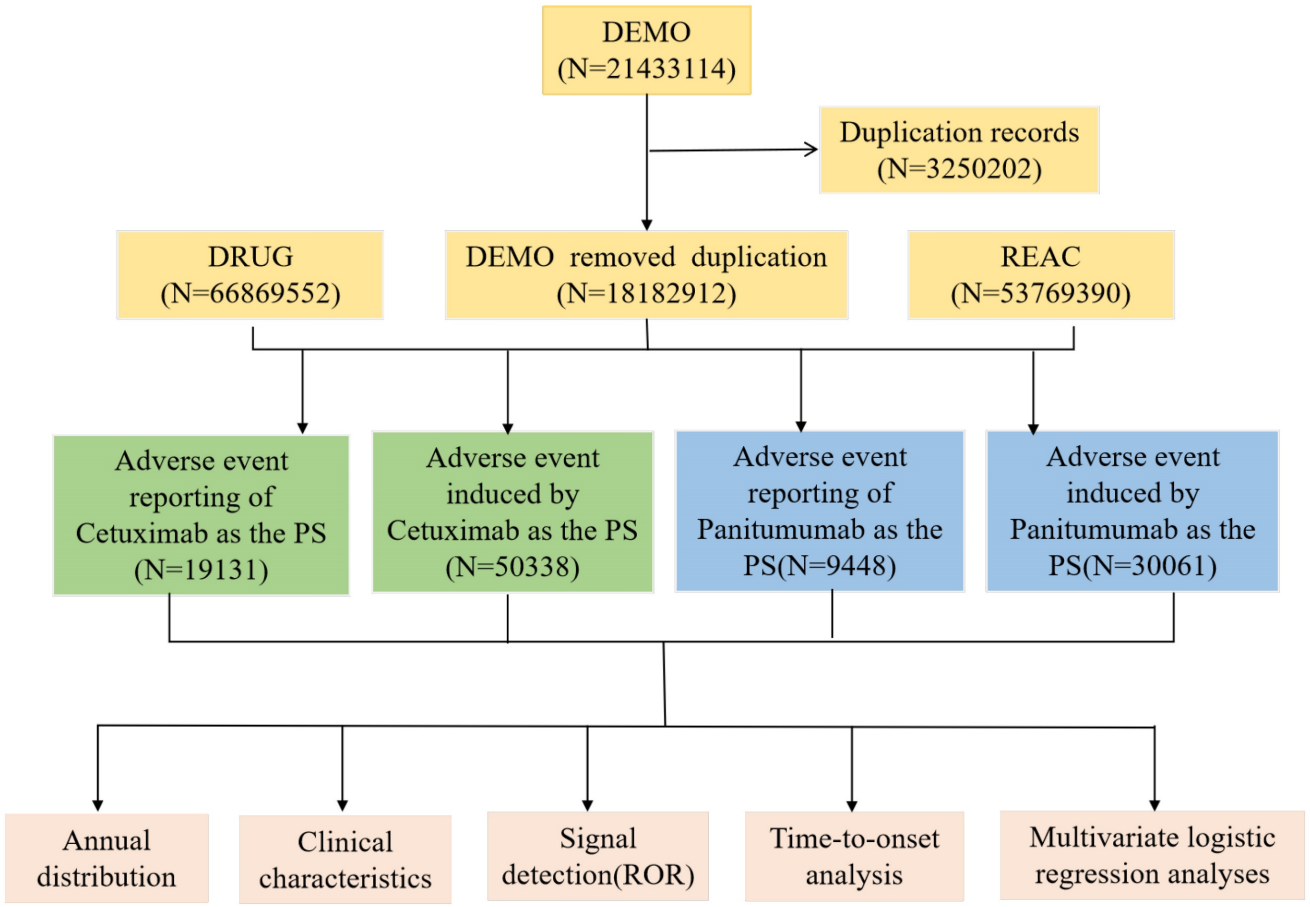
The flow diagram of two anti-EGFR mAbs-related AEs from FAERS database. Abbreviations: AEs, adverse events; EGFR, epidermal growth factor receptor; FAERS, FDA Adverse Event Reporting System; mAbs, monoclonal antibodies.

The keywords “cetuximab” and “panitumumab” or trade name were used to search the FAERS database (from Q1 2004 to Q2 2024) for all indications; only reports where cetuximab or panitumumab was identified as the primary suspect (PS) drug associated with AEs were included. All AEs were categorized using the Medical Dictionary of Regulatory Activities (MedDRA; version 25.1), and the preferred terms (PTs) were designated in accordance with systemic organ classes (SOC). MedDRA comprises five levels of increasing specificity: the lowest-level term (LLT), PT, high-level term (HLT), high-level group term (HLGT), and SOC. The screening process was illustrated in **Figure 1**. If two reports possessed identical AE identifiers, Individual Safety Report (ISR) numbers, delivery dates, medications, indications, genders, reporting nations, and age, they were deemed duplicates. The subsequent reports underwent additional screening through the designation of the PS as the main selection criterion (anti-EGFR mAbs) after probable AEs that could result from concurrent drugs and drug interactions were excluded. Following the deduplication procedure mentioned earlier, further analysis was performed utilizing the remaining reports.

### Data Analysis

For the evaluation of the signal intensity of cetuximab and panitumumab, we utilized a disproportionate analysis of reported odds ratio (ROR), Proportional Reporting Ratio (PRR), Bayesian Confidence Propagation Neural Network (BCPNN), and Empirical Bayes Geometric Mean (EBGM) methodologies, analyzing at both the SOC and PT levels. However, it revealed that the ROR method detected the greatest number of positive signals. Besides, PRR method is less sensitive to rare events, BCPNN method relies more on prior information, and EBGM method relies more on data volume. Importantly, The ROR method detected all the positive signals that were also identified by the PRR, BCPNN, and EBGM methods. In addition, ROR method is effective in identifying potential associations between drugs and AEs and has shown good performance in multiple studies^24^, ^25^. Then, we opted to use the ROR method for the following analysis. Risk of AEs was evaluated through signal detection using a disproportionality analysis, which involved calculating the ROR and its 95% CI. A risk signal was regarded as significant if the ROR and the lower boundary of the corresponding 95% CI were both greater than 1. Referencing the four-cell table of the ratio imbalance method (**Supplementary Table 1**), the formulas and criteria for these algorithms were outlined in **Supplementary Table 2**^26^. Generally, a higher algorithmic value corresponds to a more evident signal, implying a stronger correlation between the drug and the incidence of AEs. Time to onset was the period from the commencement of anti-EGFR mAb treatment to the manifestation of an AE. The time of adverse event occurrence was computed using "Time AE date - Initiation date of the use of anti-EGFR mAbs". Entries with date errors (such as when EVENT_DT comes before START_DT) or incorrect data were not included^27^. To illustrate the time to onset of AEs, The median number of days was presented along with the interquartile range, which extends from the first to the third quartile. We analyzed the signals of AEs related to anti-EGFR mAbs. This research was carried out and documented following the Standards for Quality Improvement Reporting Excellence (SQUIRE) guidelines^24^.

### Statistical analysis

We utilized multivariate logistic regression to account for age, sex, occupation of reporter, and concomitant drugs (including the top 20 concomitant drugs) when calculating the ROR. A two-tailed P-value of less than 0.05 was set as the threshold for statistical significance. R software (version 4.1.2) was used for statistical analysis and data mining.

## Results

### Clinical characteristics of the reports

We included a total of 21,433,114 AE reports in the initial screening. Once duplicate reports were removed, 19,131 cetuximab reports and 9,448 panitumumab reports remained in FAERS, which respectively resulted in 50,338 and 30,061 reported AEs. Understanding that multiple AEs were reported in a single case makes it clear why the actual number of AEs exceeds the number of reports. The process flowchart is depicted in **Figure 1**.

The relevant characteristics and clinical information are detailed in **Table 1**. Among the patients with clear gender information, the proportion of males exceeded that of females for both drugs, 58.7% vs. 27.2% for cetuximab and 51.8% vs. 30.7% for panitumumab, consistent with epidemiological findings. A small minority of participants were under 18 years and over 85 years of age, with a comparable number of patients aged 18-65 years and 66-85 years for both drugs. Among the participants with reported weight, the largest proportion (27.8% and 22.7%) were 50-100 kg for both drugs. For cetuximab, consumers reported most AEs up to 55.6%, which may be related to its tendency to cause allergies, as well as the common medical knowledge of patients and families. For panitumumab, most of the reports were filed by healthcare professionals, including physicians (56.6%), other health professionals (14.9%), health-professional (8.4%), and pharmacists (7.8%), with consumer self-reports comprising 12.1%, suggesting a reliable data source. In terms of the reporting countries, the reports on cetuximab were mainly from the United States (46.6%) and Germany (32.0%), while the reports on panitumumab were submitted in a dispersed manner, mainly from the United States (26.4%) and Japan (23.0%).

**Figure 2:**
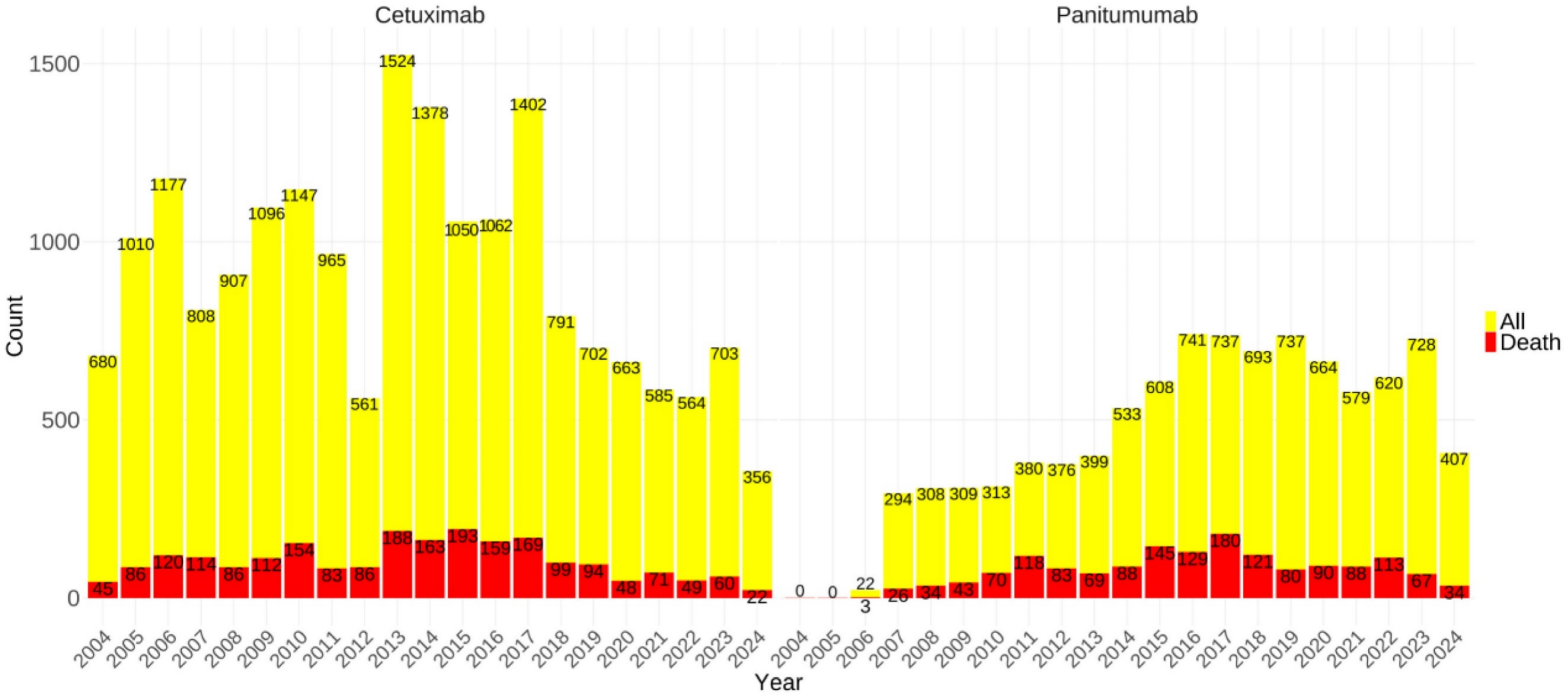
The number of reported cases of two anti-EGFR mAbs-related AEs per year. The data in 2024 were from the first half of 2024. The red bars represented the number of lethal AEs. Abbreviations: AEs, adverse events; EGFR, epidermal growth factor receptor; mAbs, monoclonal antibodies.

Furthermore, a histogram was constructed to illustrate the annual AEs metric data, thereby rendering the temporal trends more visually discernible. The incidence of AE reports with associated cetuximab exhibited significant variation over time. Specifically, the minimum number of AE reports was documented in 2012, amounting to 561 cases, whereas the highest peak was reached in 2013, with a total of 1,524 reports. Besides, the number of AEs reports for panitumumab increased relatively steadily, and the reports number on cetuximab consistently exceeds that of panitumumab. Drugs' widespread use was significantly limited by lethal adverse effects. Hence, we depicted the fatal AEs outcome metric data for both medications, indicated in red in the bar graph, as shown in **Figure 2**. Panitumumab had slightly higher proportions of fatal AEs (16.73% vs. 11.50%) as per the database reports, in comparison with cetuximab.

### Signal detects at the PT level

**Figure 3:**
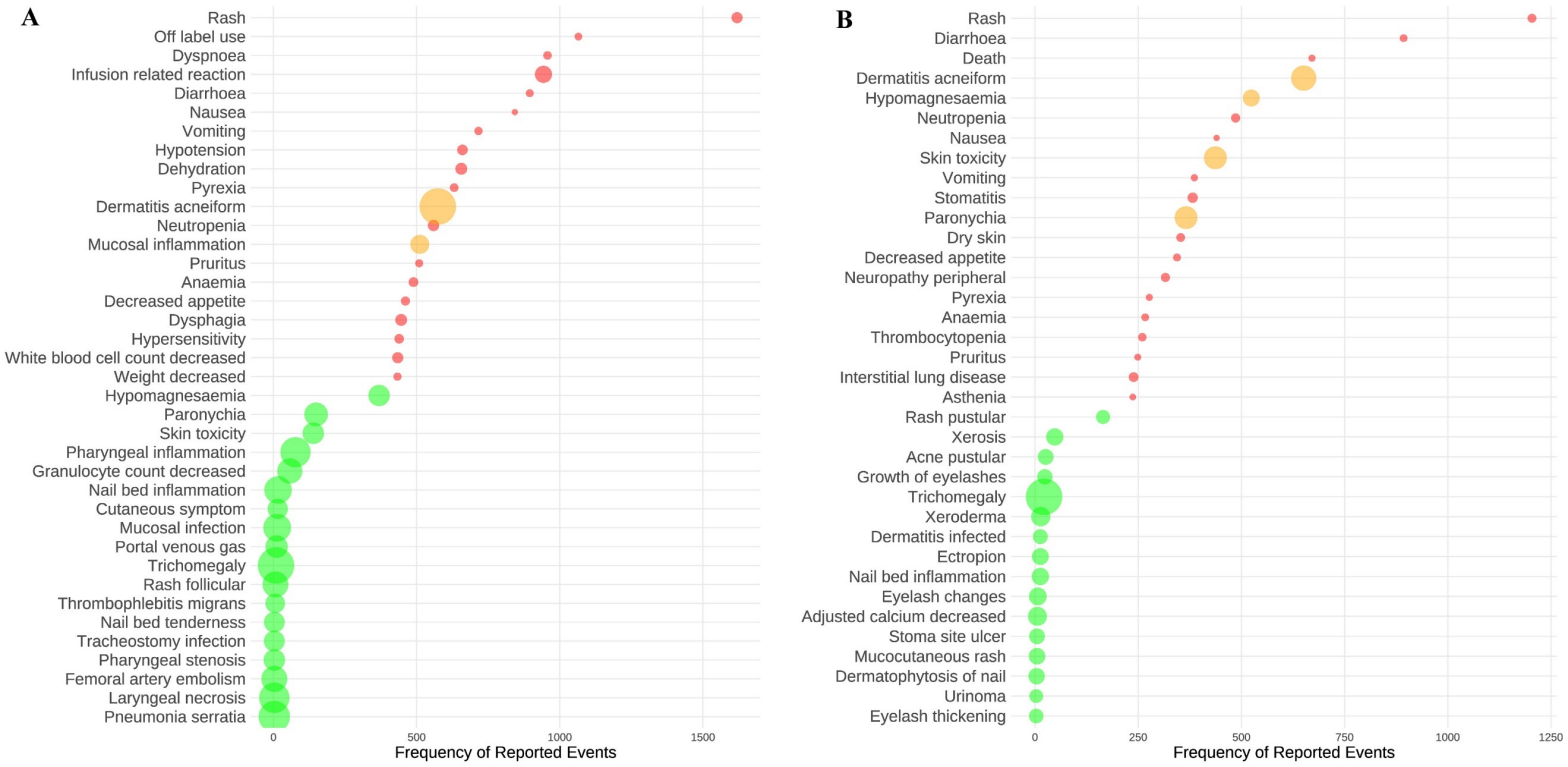
The top twenty AEs with the highest frequency and strongest signal intensity for cetuximab (A) and panitumumab (B). The AEs frequency is represented by the x-axis, the various AEs are represented by the y-axis, and the bubble size reflects the ROR value. The top twenty AEs with the highest frequency are shown in red bubbles, the top twenty AEs with the highest signal strength are shown in green bubbles, and AEs that display both high frequency and strong intensity were shown in yellow bubbles. Abbreviations: AEs, adverse events; ROR, reported odds ratio.

At the PT level, a disproportionality analysis was carried out using the ROR, with the exclusion of AEs that were evidently unrelated to the drug. This approach facilitated the detection of 574 significant AE signals associated with cetuximab and 439 with panitumumab. The analysis focused on the top twenty AEs that had the highest frequency and the most intense signals for cetuximab and panitumumab were examined. Rash was identified as the AE with the highest frequency for cetuximab (n=1,619), followed by off-label use (n=1,065) and dyspnea (n=957) (**Figure 3A**, and **Supplementary Tables 3** and** 4**). Dermatitis acneiform was identified as the AE with the highest signal intensity for cetuximab (ROR=132.56), followed by trichomegaly (ROR=131.59) and pneumonia serratia (ROR=91.48). It's worth noting that dermatitis acneiform (n=574, ROR=132.56) and mucosal inflammation (n=511, ROR=24.66) showed a notable combination of high frequency and strong signal intensity for cetuximab. Likewise, as shown in** Figure 3B**, and **Supplementary Tables 3** and** 4**, rash was identified as the AE with the highest frequency in panitumumab treatment (n=1204), followed by diarrhea (n=893) and death (n=671). The AEs with the most intense signal for panitumumab were trichomegaly (ROR=655.96), dermatitis acneiform (ROR=258.74), and skin toxicity (ROR=198.52). Moreover, dermatitis acneiform (n=651, ROR=258.74), hypomagnesaemia (n=524, ROR=83.35), skin toxicity (n=437, ROR=198.52), and paronychia (n=366, ROR=197.47) showed both high frequency and most intense signal for panitumumab.

Given the considerable overlap in AEs observed with cetuximab and panitumumab, a detailed comparative analysis of the convergent AE profiles was undertook. In the analysis of the 215 identical positive AE signals between the two drugs, 83 AEs of cetuximab were found to have a stronger correlation than panitumumab, while 132 AEs of cetuximab had a weaker correlation than panitumumab, based on the ROR value. **Table 2** provided a comparison of the AE signals between the two drugs, revealing significant differences in intensity. The AE signals of cetuximab were found to have a stronger correlation than panitumumab (d > 10), in order of preference, included rash follicular (ROR: 56.6 vs. 39.44), infusion-related reaction (ROR: 18.25 vs. 3.4), and cutaneous symptom (ROR: 29.38 vs. 16.11), suggesting that cetuximab may have a higher tendency toward these AEs than panitumumab. In contrast, panitumumab's AE signals with a stronger correlation than cetuximab (d > 30) were linked to trichomegaly (ROR: 655.96 vs. 131.59), skin toxicity (ROR: 198.52 vs. 34.66), and paronychia (ROR: 197.47 vs. 44.66). The explanation could be that, despite their seeming similarities, these two mAbs differ significantly in terms of biology, molecular structure, and application.

### Signal detects at the SOC level

**Figure 4:**
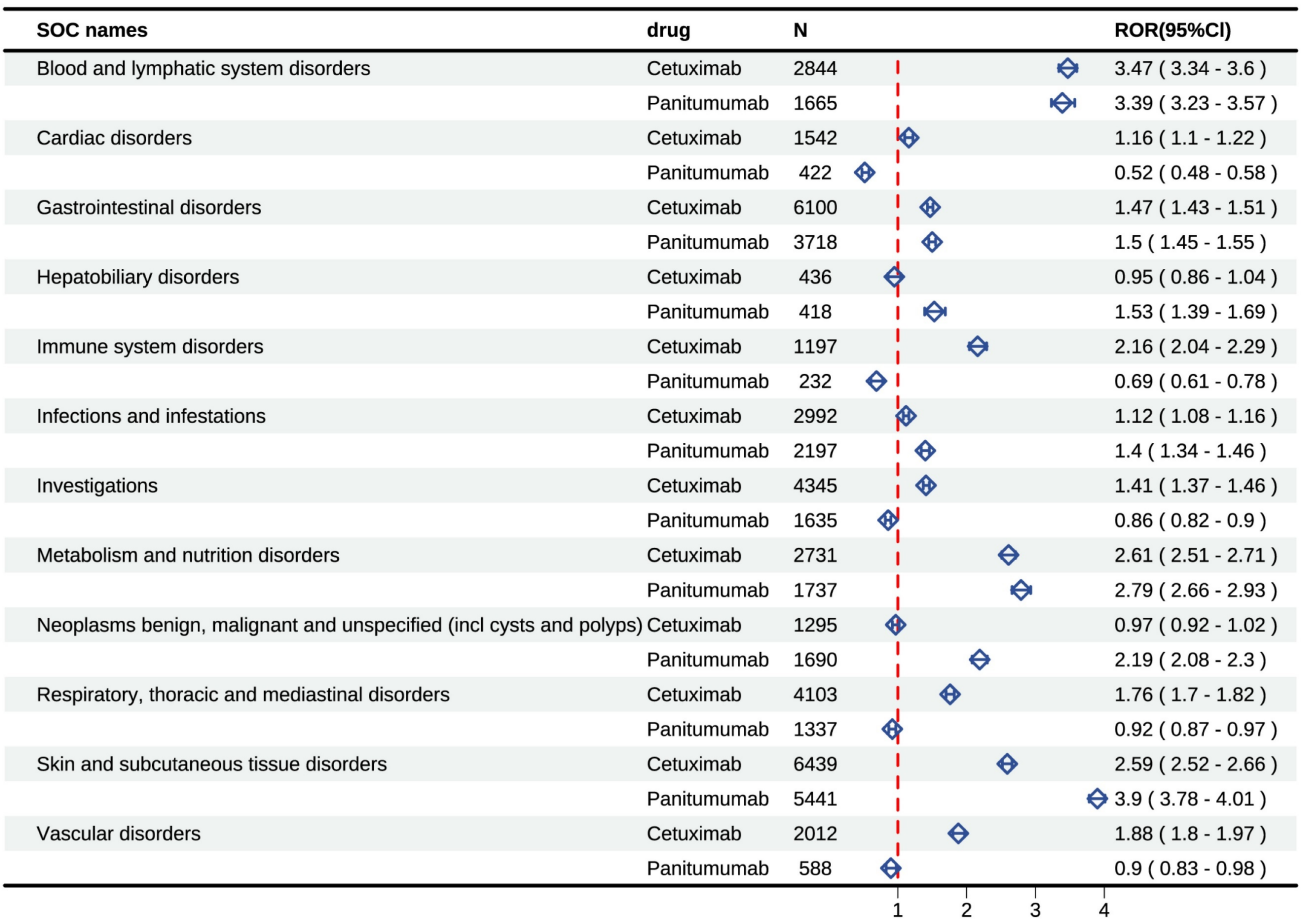
Cetuximab and panitumumab-related significant AEs at the SOC level. Abbreviations: AEs, adverse events; SOC, systemic organ classes.

To provide a more nuanced understanding of the distinctions between cetuximab and panitumumab, we comprehensively presented the signal mining results at the SOC level (**Figure 4** and **Supplementary Table 5**). Our statistical analysis indicated that the occurrence of AEs induced by cetuximab was largely focused on 24 SOCs, providing insights into the specific systems affected. Several significant SOCs were identified among these, with a case number of three or more, highlighting areas of particular interest for further investigation, for which the ROR algorithm met the criteria. The study found that cetuximab was associated with a total of 10 positive signals at the SOC level, providing insights into its overall safety profile, of which “blood and lymphatic system disorders” displayed the most powerful signal (ROR=3.47), followed by “metabolism and nutrition disorders” (ROR=2.61), as well as “skin and subcutaneous tissue disorders” (ROR=2.59). Other serious AEs included “immune system disorders” (ROR=2.16), “vascular disorders” (ROR=1.88), “respiratory, thoracic, and mediastinal disorders” (ROR=1.76), “gastrointestinal disorders” (ROR=1.47), etc. Additionally, our statistical analysis also revealed that 23 SOC were the main focus of panitumumab-induced adverse events. Of them, several positive SOC were identified, for which the ROR algorithm met the criteria. Panitumumab involved 7 positive signals at the SOC level, including “skin and subcutaneous tissue disorders” (ROR=3.9), “blood and lymphatic system disorders” (ROR=3.39), as well as “metabolism and nutrition disorders” (ROR=2.79). In addition, other rare signals included “neoplasms benign, malignant, and unspecified, hepatobiliary disorders”, “gastrointestinal disorders”, as well as “infections and infestations”.

### Time to onset analyses across different SOC dimensions

**Figure 5:**
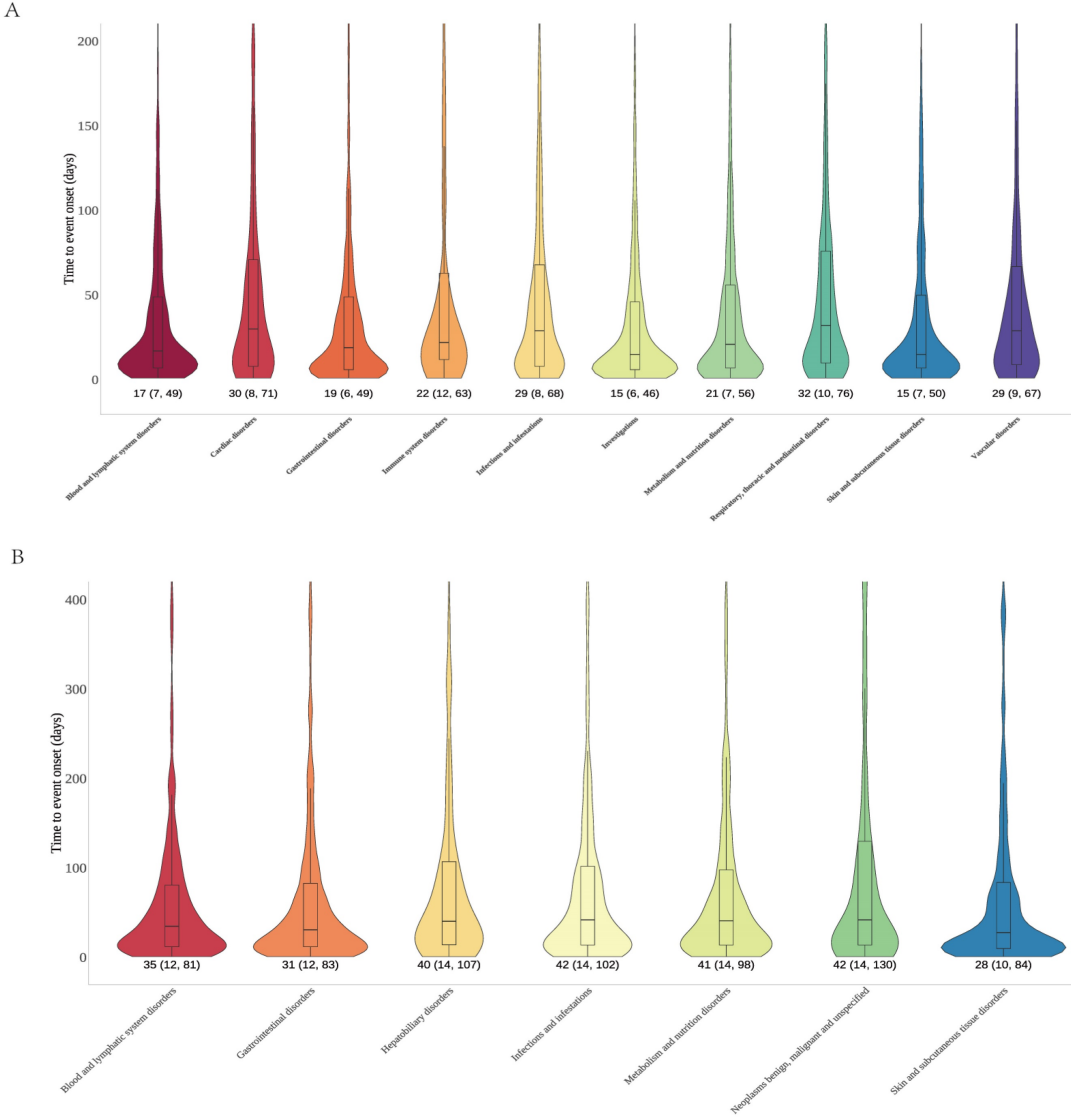
Time to onset of significant signal at the SOC level for cetuximab (A) and panitumumab (B). Abbreviations: SOC, systemic organ classes.

Time to onset analyses for cetuximab and panitumumab were performed based on the different SOC that exhibited positive signals, in order to elucidate the temporal patterns of the AEs. We plotted onset time distribution of AEs for two drugs, as shown in **Figure 5**. Cetuximab involved 10 positive signals; the time of onset varies across SOC, but it showed that substantial number of AEs manifested within 30 days. The SOC of skin/subcutaneous tissue disorders and the SOC of investigations both exhibited a median time to onset of 15 days, which was the earliest (IQR: 7, 50 and IQR: 6, 46). Conversely, the SOC of respiratory, thoracic, and mediastinal disorders showed the greatest median time to onset was 32 days (IQR: 10, 76). Furthermore, the median time to onset for the blood and lymphatic system disorders SOC was 17 days (IQR: 7, 49), and for gastrointestinal disorders was 19 days (IQR: 6, 49), etc. Panitumumab involved 7 positive signals; the skin/subcutaneous tissue disorders exhibited the earliest median duration to onset of 28 days (IQR: 10, 84), followed by gastrointestinal disorders of 31 days (IQR: 12, 83) as well as blood and lymphatic system disorders of 35 days (IQR: 12, 81). In addition, neoplasms benign, malignant and unspecified as well as infections and infestations SOC both showed the greatest median duration to onset of 42 days (IQR: 14, 130 and IQR: 14, 102).

### Sensitivity analysis

To explore potential associations between the incidence of AEs and various factors such as age, sex, reporter occupation, and concurrent medications (**Supplementary Table 6**), logistic regression models, both univariate and multivariate, were constructed. The specific model parameters are detailed in **Table 3**. Four distinct model strategies were applied to boost the robustness of the results. The outcome demonstrated a strong statistical correlation between the target drugs and AEs. After adjusting for confounding factors, panitumumab demonstrated a robustly higher risk of AEs compared with cetuximab.

## Discussion

Within the treatment scenario of mCRC, cetuximab and panitumumab are presently employed as second- or third-line therapeutic options^28^. As far as we are aware, this investigation offers a pioneering exploration into the comprehensive pharmacovigilance analysis of AEs associated with cetuximab and panitumumab in the phase following market approval.

The findings of our research indicated that male patients exhibited a higher incidence of AEs associated with cetuximab and panitumumab. This observation might result from the higher prevalence of CRC in this demographic subgroup^6^. A substantial majority of panitumumab-related reports originate from healthcare professionals, indicating a potentially more reliable reporting source. Conversely, cetuximab-related cases are predominantly reported by patients themselves. This discrepancy may be attributed to the more pronounced adverse events associated with skin and subcutaneous tissue disorders for cetuximab, which are readily noticeable by patients. It is noteworthy that the preponderance of reported cases emanated from the United States. This trend may be ascribed to the substantial volume of pharmaceutical utilization within the country, coupled with the heightened emphasis placed on the documentation of adverse drug events. Moreover, the incidence of AE reporting can exhibit variability not only across different medications but also for the same drug over successive time intervals. The year-wise spread of reports may have been affected by the specific timing of cetuximab's and panitumumab's approval and subsequent clinical use, suggesting a correlation between these events and reporting trends. The varying upward trend of reported AEs linked to both medications across the 20 years that FAERS has been operating highlights the need for epidemiological research. Whether arranged by number of cases reported or signal strength, cetuximab and panitumumab share a large number of overlapping AEs. The identified AEs in this analysis were generally in agreement with the known AEs of these drugs, indicating the study's validity and suggesting that the findings may provide an accurate reflection of real-world clinical practices, thereby reinforcing the reliability of the results.

The key differences of SOC between the two anti-EGFR mAbs included the likelihood of “skin and subcutaneous tissue disorders”, “vascular disorders”, “respiratory, thoracic, and mediastinal disorders”, “cardiac disorders”, “hepatobiliary disorders”, “immune system disorders”, and “neoplasms benign, malignant, and unspecified (incl cysts and polyps)”. Although cetuximab and panitumumab fall under the same category, there were notable differences in the AEs associated with each drug. The ROR signal intensity linked to cetuximab at the SOC level was significantly higher than that of panitumumab when considering “cardiac diseases”, “immune system disorders”, “respiratory, thoracic, and mediastinal disorders” and “vascular disorders”, was significantly higher than that of panitumumab. At the PT level, similar results were seen, including fatal adverse events like interstitial lung disease (ILD) and cardio-respiratory arrest which are severe AEs related to “respiratory, thoracic and mediastinal disorders” and “cardiac diseases”, aligning with the SOC-level results. Compared with cetuximab, panitumumab had stronger signal intensity of ROR associated with “hepatobiliary disorders”, “infections and infestations”, “neoplasms benign, malignant and unspecified (incl cysts and polyps)” and “skin and subcutaneous tissue disorders”. The results are in line with the safety information listed in the label and clinical trials. However, the SOC of “hepatobiliary disorders” of panitumumab is a novel signal, which has not been noted in prior studies.

It is reported that around 80% of patients receiving anti-EGFR mAbs treatment develop skin toxicity, with approximately 10% of cases classified as grade 3-4 toxicity^29^. Patients may experience skin toxicity ranging from dry skin to a papulo-pustular rash, which is often described as “acnelike” or “acneiform” rash. Our research showed that dermatitis acneiform was identified as the most common AE for cetuximab and panitumumab, and in the skin and subcutaneous tissue systems, it was characterized by high frequency and strong signal intensity (**Figure 3**). The incidence of grade 3-4 skin and subcutaneous tissue toxicities was similar between the panitumumab and cetuximab groups, according to a large phase III clinical study^21^. In our study, we observed that the overall signals at the SOC level were stronger for panitumumab compared to cetuximab in skin and subcutaneous tissue systems (ROR: 3.9 vs. 2.59), including rash (ROR: 5.97 vs. 4.76), dermatitis acneiform (ROR: 258.74 vs. 132.56), skin toxicity (ROR: 198.52 vs. 34.66) and dry skin (ROR: 5.79 vs. 3.05). Further, our research discovered that cetuximab exhibited the earlier median time to onset than panitumumab in skin and subcutaneous tissue systems (15 days vs. 28 days) (**Figure 5**).

Although both cetuximab and panitumumab exerted adverse effects on the skin, the clinical manifestations of these dermatologic symptoms exhibited distinct profiles. Although the physiopathology of this toxicity is poorly understood, the high expression of EGFR in the outer layers of hair follicles and the basal layers of the epidermis may help to explain it^30^. It has been demonstrated that EGFR activation regulates normal keratinocyte proliferation, differentiation, migration, and survival, whereas EGFR suppression resulted in growth and migratory arrest, inflammatory cytokine release, immune cell recruitment, and apoptosis^31^. According to a recent dermopathology study, most anti-EGFR drugs, including cetuximab, panitumumab, erlotinib, and lapatinib, induce epidermal atrophy. However, panitumumab and cetuximab seem to be especially associated with dermal immune cell infiltration. This observation aligns with the hypothesis that cetuximab's activity is partially mediated by ADCC. Specifically, mononuclear cells were predominantly found in skin lesions associated with cetuximab, whereas neutrophils and neutrophilic pustules were the main characteristics of skin lesions induced by panitumumab^32^. This also perfectly explains the longer onset of panitumumab in the skin/subcutaneous tissue system as well as the results in **Table 2** in this study; the AEs of dermatitis acneiform and acne pustular for panitumumab were obviously stronger than cetuximab (d > 30). This result, which complements the safety results of previous clinical studies, is of great significance in guiding internists in the organization of clinical treatment protocols.

Hypersensitivity reactions were predominantly associated with cetuximab rather than panitumumab. Infusion-related reactions posed a potentially life-threatening risk for cetuximab users, prompting an FDA “black box” warning and recommendations to permanently discontinue use in patients with grade ≥3 infusion-related reactions. Our study revealed that infusion reactions (n=943, ROR=18.25) and hypersensitivity (n=439, ROR=2.87) associated with cetuximab were highly prevalent and prominently featured among the frequently documented AEs. In contrast, the occurrence of such events with panitumumab was significantly lower. Likewise, among comparative analyses to evaluate the strength of AE signals between these two drugs, compared with panitumumab, AE signals of infusion-related reactions with ROR values showing significant differences between cetuximab and panitumumab (d > 10). Panitumumab has shown a correlation with 0% to 3% grade 3 infusion reactions, and no fatal reactions have been documented to date^33^. A retrospective study comprising 51 patients by LaPlant *et al.* revealed minimal influence of different pretreatment schemes on hypersensitivity reaction rates. However, this study demonstrated that panitumumab could be safely administered to patients with severe and refractory hypersensitivity reactions to cetuximab^34^. The primary pharmacological difference between the drugs is in their IgG backbone - panitumumab does not require premedication, and the incidence of hypersensitivity is lower because of the fully humanized nature of the antibody.

An active transport process helps facilitate magnesium reabsorption in the distal convoluted tubule, while recent evidence has highlighted that EGF plays a regulatory role in modulating this mechanism^35^. Consequently, hypomagnesemia may ensue due to diminished renal reabsorption of magnesium, a consequence of EGFR inhibition. In our study, hypomagnesaemia (n=524, ROR=83.35) had both high frequency and pronounced signal intensity for panitumumab; the ROR signals were higher than for cetuximab. A phase 3 head-to-head study highlighted that the incidence of grade 3-4 hypomagnesaemia was greater among patients receiving panitumumab (35 [7%] patients) compared to those on cetuximab (13 [3%] patients). Furthermore, hypomagnesaemia prompted discontinuation of study treatment in six (1%) patients in the panitumumab group and two (<0.5%) in the cetuximab group. Owing to hypomagnesaemia, dose modifications were necessary for 25 (5%) patients in the panitumumab group and 14 (3%) in the cetuximab group^21^. This result is consistent with our findings and confirms the validity of our study. This could be because, despite the fact that both drugs are monoclonal antibodies that target the EGFR, their affinities for the receptor differ; panitumumab may have a higher affinity and be more effective at blocking the receptor, which could have a greater impact on magnesium reabsorption and enhance the risk of hypomagnesemia. It is advised that patients receiving anti-EGFR mAb therapy monitor magnesium metrics and benefit from intravenous and/or oral magnesium supplements when needed.

Elucidating the thromboembolic risk associated with pharmacotherapy is of paramount importance, given that cancer patients inherently exhibit a hypercoagulable state, which may be further exacerbated by drug exposure, thereby increasing the likelihood of thromboembolic events. In our analysis, panitumumab was a negative signal (**Figure 4**), while cetuximab-associated vascular diseases were a positive signal at the SOC level (n=2012, ROR=1.88). Furthermore, cetuximab was found to cause a wide range of thromboembolism events at the PT level, including deep vein thrombosis (n=198, ROR=3.53), thrombosis (n=95, ROR=1.39), embolism (n=31, ROR=4.35), venous thrombosis (n=20, ROR=6.09), and others. Although the number of thromboembolic events linked to panitumumab was small and the signal strength was low, it was nevertheless significant. For example, embolism (n=21, ROR=4.93), venous thrombosis (n=16, ROR=8.16), infarction (n=8, ROR=2.47), deep vein thrombosis (n=71, ROR=2.11), etc. A meta-analysis showed that cancer patients on anti-EGFR monoclonal antibody-containing therapies are roughly 1.5 times more likely to experience venous or pulmonary embolism compared to those receiving the same therapies without anti-EGFR mAbs^20^. Various processes have been implicated in explaining the hypercoagulable state seen in cancer patients on anticancer drug therapy. Specifically,* in vitro* experiments have revealed that human microvascular endothelial cells exposed to EGF exhibit augmented plasminogen activator expression, offering insights into this condition^36^. However, it is more likely that anti-EGFR mAbs may impede the identification of endothelial damage resulting from co-administered agents, thus facilitating platelet activation, leukocyte adhesion, oxidative stress, coagulation, and inflammation—all of which are integral to thromboembolism development. When selecting treatment regimens containing cetuximab or panitumumab, clinicians should consider the patient's baseline thromboembolic risk.

Although ILD occurs infrequently, it represents a potentially life-threatening adverse effect of anti-EGFR mAb that warrants vigilant attention and thorough investigation. The overall signals for cetuximab of "respiratory, thoracic, and mediastinal disorders" were greater than those for panitumumab; nevertheless, at the PT level, panitumumab showed a robust signal in the ILD (n=239, ROR=10.33) and pneumonitis (n=43, ROR=3.38) (**Supplementary Table 5**). For ILD (n=220, ROR=5.65) and pneumonitis (n=133, ROR=6.28), cetuximab exhibited a strong signal, but the ROR was significantly lower than that of panitumumab. This could be explained by the fact that EGF controls the immune and inflammatory responses by inhibiting the chemotaxis of alveolar macrophages and maintaining the regeneration and repair of airway epithelial cells. By inhibiting EGFR activation in malignant tissues and stopping the growth of airway epithelial cells, anti-EGFR medications may damage lung tissue. It is highly recommended to maintain a high level of ILD surveillance throughout clinical practice and to quickly identify those individuals who are at a heightened risk^37^.

Cardiotoxicity, a common adverse effect associated with numerous cancer therapeutic agents, significantly jeopardizes patient safety and adversely impacts prognosis^38^, ^39^. Our research indicated that cetuximab showed a higher proportion of AEs than panitumumab. The most common cardiotoxicity events associated with cetuximab were tachycardia (ROR=2.56), cardiac arrest (ROR=2), and cardio-respiratory arrest (ROR=4.69). Cardiopulmonary failure (ROR=2.98) was the single cardiotoxicity incident linked to panitumumab. It suggested that 38.7% of patients appeared to have abnormal electrocardiograms with transient and reversible ST depression and the elevation of troponin I ultra during cetuximab therapy^40^. This is supported by our data, which did not include significant cardiotoxic side effects, including QT interval prolongation. Despite their low cardiotoxicity, cetuximab and panitumumab still warrant baseline cardiac assessment and appropriate monitoring.

Ocular toxicity, though infrequently encountered as a drug-induced AE, can profoundly impair quality of life. Eye irritation (ROR=1.56) was the most frequently encountered ocular AE for panitumumab. The ocular hyperaemia (ROR=1.58), growth of eyelashes (ROR=59.22), and trichomegaly (ROR=655.96) for panitumumab, as well as periorbital edema (ROR=2.59) and growth of eyelashes (ROR=14.44) for cetuximab, were not mentioned in the instruction. Still, serous retinal detachment AEs indicated that ocular toxicity should not be underestimated.

Our analysis revealed a relatively low incidence of reported hepatobiliary disorder cases for both cetuximab and panitumumab. The analysis indicated a higher proportion of hepatobiliary AEs related to panitumumab compared to cetuximab. These cases primarily showed up as jaundice, hyperbilirubinemia, cholangitis, hepatic function abnormalities, and hepatic failure. The hepatic failure (n=53, ROR=3.51), hepatic function abnormal (n=39, ROR=2.18), and jaundice (n=36, ROR=2.61) for panitumumab were not mentioned in the instruction. Given that this study first identified panitumumab-related hepatobiliary adverse events, it is recommended that baseline liver function tests be completed prior to initiating panitumumab therapy. During the initial treatment period, ALT, AST, total bilirubin (TBil), and alkaline phosphatase (ALP) levels should be monitored every 2 weeks. Our discovery that panitumumab-induced hepatobiliary illness is a unique signal that has not been documented in the specification or in prior research is extremely clinically significant.

Panitumumab treatment was found to be associated with a wide array of neurologic AEs, including peripheral neuropathy (n=316, ROR=7.1), peripheral sensory neuropathy (n=96, ROR=34.83), polyneuropathy (n=81, ROR=14.2), neurotoxicity (n=66, ROR=8.28), dysgeusia (n=54, ROR=1.4), and acute polyneuropathy (n=3, ROR=25.79). Based on the ROR signals, there might be a greater incidence of neurotoxicity among patients receiving panitumumab than cetuximab, which is consistent with the study of the analysis in neuronal toxicity of monoclonal antibodies by Nitin Kumar *et al.*^41^. To minimize the occurrence of neurotoxicity, it is recommended that baseline examinations be completed prior to panitumumab administration, followed by assessments using the Common Terminology Criteria for Adverse Events (CTCAE v5.0) every 2-3 treatment cycles to document symptoms. Neuroprotective interventions should be initiated as necessary.

Our research recognizes several limitations that need to be addressed. Firstly, the inherent confounding factors in real-world data may affect the quantitative distortions, which might influence the detection of full AE signals. Secondly, the geographic concentration of the reports is mainly from the United States and Europe, which may restrict the applicability to all over the world populations. Thirdly, we are unable to observe the basic chemotherapy regimens, which requires large-scale global multi-center studies. Fourthly, the lack of detailed exposure data precluded us from estimating the morbidity and mortality rates. Finally, owing to the inherent structure of the FAERS database, detailed tumor characteristics such as TNM stage, metastatic burden, combination chemotherapy regimens, and primary tumor location were unavailable. Consequently, tumor staging or combination chemotherapy regimens could not be incorporated into the multivariate logistic regression. Future prospective cohort studies with complete clinical annotation are warranted to further elucidate the interaction between tumor stage or combination chemotherapy regimens and anti-EGFR monoclonal antibody-associated adverse events.

Future research must explore uncharted domains to advance our understanding and application of anti-EGFR monoclonal antibodies in cancer therapy. Firstly, conduct large-scale, multinational prospective cohort studies to develop stratified surveillance guidelines based on real-world evidence; Secondly, given the genetic heterogeneity among colorectal cancer patients, implement pharmacogenomics-driven personalized risk management to explore gene-drug interactions and identify genetic markers predictive of individual adverse event risks, thereby providing evidence for future proactive pharmacogenomic screening; Thirdly, conduct in-depth research on preclinical models guided by adverse drug reaction mechanisms to identify early biomarkers for use as surrogate endpoints in clinical trials; Finally, conduct real-world comparative effectiveness studies based on combination therapy strategies, focusing on the risk of cumulative adverse events and dose adjustment patterns to inform clinical practice. For instance, skin toxicity from anti-EGFR mAbs is common but not fully understood. Elucidating these mechanisms can lead to targeted interventions to reduce AEs. Lastly, combination therapies, such as pairing anti-EGFR mAbs with immunotherapy or targeted therapy, deserve more attention. This approach may boost efficacy and lower AE risk. In summary, our study offers a thorough real-world analysis of cetuximab and panitumumab safety profiles, highlighting their differences and offering clinical insights. But enhancing cancer treatment is an ongoing quest. By adopting new research methods and technologies, and promoting cross-disciplinary collaboration, we can keep advancing our knowledge and improving CRC patient outcomes.

## Conclusion

This research holds substantial importance for post-marketing surveillance, improving the capacity to effectively monitor and manage potential AEs associated with the antitumor agents panitumumab and cetuximab. Future studies should explore the mechanisms behind AEs and concentrate on creating targeted prevention strategies. Such endeavors are crucial for enhancing the quality of life and overall outcomes for tumor individuals using these medications.

## Supplementary Material

Supplementary tables.

## Figures and Tables

**Table 1:** Demographic characteristics of AEs reported with cetuximab and panitumumab in the FAERS database.

Characteristics	Cetuximab (N = 19131)	Panitumumab (N = 9448)
**Gender**		
Female	5201 (27.2%)	2905 (30.7%)
Male	11227 (58.7%)	4897 (51.8%)
Unknown	2703 (14.1%)	1646 (17.4%)
**WT / kg**		
<50 kg	764 (4.0%)	327 (3.5%)
>100 kg	527 (2.8%)	151 (1.6%)
50∼100 kg	5309 (27.8%)	2140 (22.7%)
Unknown	12531 (65.5%)	6830 (72.3%)
**Age / years**		
<18	16 (0.1%)	47 (0.5%)
18∼65	6907 (36.1%)	2838 (30.0%)
66∼85	5911 (30.9%)	2619 (27.7%)
>85	152 (0.8%)	47 (0.5%)
Unknown	6145 (32.1%)	3897 (41.2%)
**Occupation of reporters**		
Consumer	10633 (55.6%)	1139 (12.1%)
Health-Professional	249 (1.3%)	797 (8.4%)
Lawyer	2 (0.0%)	2 (0.0%)
Physician	2147 (11.2%)	5343 (56.6%)
Other health-professional	4811 (25.1%)	1408 (14.9%)
Pharmacist	928 (4.9%)	740 (7.8%)
Registered Nurse	14 (0.1%)	2 (0.0%)
Unknown	347 (1.8%)	17 (0.2%)
**Reported countries (Top 5)**		
1	US 8909(46.6%)	US 2496(26.4%)
2	DE 4536(32.0%)	JP 2177(23.0%)
3	JP 736(3.8%)	FR 546(5.8%)
4	CN 361(1.9%)	DE 496(5.2%)
5	FR 256(1.3%)	CO 481(5.1%)

Abbreviations: AEs, adverse events; CA, Canada; CN, China; CO, Colombia; DE, Germany; FAERS, FDA Adverse Event Reporting System; FR, France; GB, United Kingdom; JP, Japan; US, United States.

**Table 2:** AEs signals with significant differences in ROR values between cetuximab and panitumumab.

Items	AEs	ROR (95% CI) for cetuximab	ROR (95% CI) for panitumumab
**AEs signals of cetuximab stronger than panitumumab (d>10)**	Rash follicular	56.6 ( 26.46 - 121.06 )	39.44 ( 12.56 - 123.82 )
	Infusion related reaction	18.25 ( 17.1 - 19.47 )	3.4 ( 2.81 - 4.1 )
	Cutaneous symptom	29.38 ( 17.59 - 49.08 )	16.11 ( 6.68 - 38.86 )
	Myelosuppression	15.23 ( 13.45 - 17.26 )	2.69 ( 1.84 - 3.92 )
	Mucosal inflammation	24.66 ( 22.58 - 26.93 )	12.19 ( 10.4 - 14.29 )
	Pneumatosis intestinalis	18.89 ( 13.92 - 25.63 )	7.43 ( 3.99 - 13.83 )
**AEs signals of panitumumab stronger than cetuximab (d>30)**	Trichomegaly	131.59 ( 65.84 - 263.01 )	655.96 ( 402.4 - 1069.28 )
	Skin toxicity	34.66 ( 29.27 - 41.05 )	198.52 ( 179.73 - 219.27 )
	Paronychia	44.66 ( 37.9 - 52.62 )	197.47 ( 177.17 - 220.1 )
	Dermatitis acneiform	132.56 ( 121.49 - 144.62 )	258.74 ( 238.16 - 281.1 )
	Xerosis	4.16 ( 1.56 - 11.1 )	87.43 ( 65.42 - 116.84 )
	Acne pustular	13.18 ( 6.83 - 25.43 )	65.34 ( 44.17 - 96.65 )
	Hypomagnesaemia	34.22 ( 30.84 - 37.97 )	83.35 ( 76.3 - 91.04 )
	Growth of eyelashes	14.44 ( 7.74 - 26.96 )	59.22 ( 39.43 - 88.96 )
	Dermatitis infected	9.64 ( 3.6 - 25.79 )	53.57 ( 30.85 - 93.02 )
	Peripheral sensory neuropathy	3.83 ( 2.41 - 6.08 )	34.83 ( 28.45 - 42.64 )

Note: d, difference of ROR, between cetuximab and panitumumab. Abbreviations: AEs, adverse events; ROR, reported odds ratio.

**Table 3:** Univariate and multivariate logistic regression analyses of adverse events associated with cetuximab and panitumumab.

Drugs	ROR(95% CI)			
	Model 1	Model 2	Model 3	Model 4
Cetuximab	Reference	Reference	Reference	Reference
Panitumumab	1.55 (1.44, 1.66)*	1.53 (1.41, 1.67)*	1.55 (1.41, 1.70)*	1.56 (1.41, 1.72)*

The asterisk indicates statistical significance. Model 1: crude ROR. Model 2: adjusted for age and sex. Model 3: adjusted for age, sex, and occupation of reporter. Model 4: adjusted for age, sex, occupation of reporter, and concomitant drugs (included top 20 concomitant drugs). Abbreviations: ROR, reported odds ratio.

## Abbreviations

AEs: adverse events
BCPNN: Bayesian Confidence Propagation Neural Network
CRC: colorectal cancer
EBGM: empirical Bayesian geometric mean
EGFR: epidermal growth factor receptor
FAERS: FDA Adverse Event Reporting System
HLGT: high-level group term
HLT: high-level term
ILD: interstitial lung disease
IQR: interquartile range
LLT: lowest-level term
mAbs: monoclonal antibodies
mCRC: metastatic colorectal cancer
MedDRA: Medical Dictionary of Regulatory Activities
PRR: Proportional Reporting Ratio
PS: primary suspect
PTs: preferred terms
ROR: reported odds ratio
SOC: systemic organ classes
SQUIRE: Standards for Quality Improvement Reporting Excellence
