# Supplementary material for: Real-World Safety of Anti-EGFR Antibodies: 20-Year Pharmacovigilance of Cetuximab and Panitumumab
Source: Int J Med Sci. 2025 Sep 29;22(15):4131–44. doi: 10.7150/ijms.122194 (PMC12492352; doi:10.7150/ijms.122194)
Supplement: Supplementary file 1 — Supplementary tables. [file ijmsv22p4131s1.pdf]

Supplementary Table 1. Four-cell table of proportional disequilibrium method.

**Drugs**	**Target adverse reaction**	**Other adverse reactions**	**Total**
Target drug	a	b	a+b
Other drugs	c	d	c+d
Total	a+c	b+d	n=a+b+c+d

a: the number of reports containing both the suspect drug and the suspect adverse drug reaction; b: the number of reports containing the suspect adverse drug reaction with other medications (except the drug of interest); c: the number of reports containing the suspect drug with other adverse drug reactions (except the event of interest); d: the number of reports containing other medications and other adverse drug reactions; n: the number of all reports.

Supplementary Table 2. Four major algorithms used for signal detection.

Algorithms	Equation	Criteria
ROR	ROR=ad/b/c	Lower limit of 95% CI>1, a≥3
	95%CI=$e^{\ln(ROR)\pm1.96(1/a+1/b+1/c+1/d)^{\wedge}0.5}$	
PRR	PRR=a(c+d)/c/(a+b)	PRR≥2, χ^2≥4, N≥3
	χ^2=[(ad-bc)^2](a+b+c+d)/[(a+b)(c+d)(a+c)(b+d)]	
BCPNN	IC=$\log_2$a(a+b+c+d)/[(a+c)(a+b)]	IC025>0
	95%CI= E(IC) ± 2V(IC)^0.5	
MGPS	EBGM=a(a+b+c+d)/[(a+c)(a+b)]	EBGM05>2
	95%CI=$e^{\ln(EBGM)\pm1.96(1/a+1/b+1/c+1/d)^{\wedge}0.5}$	

Equation: a, number of reports containing both the target drug and target adverse drug reaction; b, number of reports containing other adverse drug reaction of the target drug; c, number of reports containing the target adverse drug reaction of other drugs; d, number of reports containing other drugs and other adverse drug reactions. 95%CI, 95% confidence interval; N, the number of reports; X^2, chi-squared; IC, information component; IC025, the lower limit of 95% CI of the IC; E(IC), the IC expectations; V(IC), the variance of IC; BCPNN, Bayesian Confidence Propagation Neural Network; EBGM, empirical Bayesian geometric mean; EBGM05, the lower limit of 95% CI of EBGM; PRR, Proportional Reporting Ratio.

Supplementary Table 3. The top twenty AEs with the highest frequency for cetuximab and panitumumab.

Drug	PTs	Frequency	SOC	ROR (95% Cl)
**Cetuximab**	rash	1619	skin and subcutaneous tissue disorders	4.76 (4.53 - 5)
	off label use	1065	injury, poisoning and procedural complications	1.6 (1.5 - 1.7)
	dyspnoea	957	respiratory, thoracic and mediastinal disorders	2.05 (1.92 - 2.19)
	infusion related reaction	943	injury, poisoning and procedural complications	18.25 (17.1 - 19.47)
	diarrhoea	895	gastrointestinal disorders	1.7 (1.59 - 1.82)
	nausea	843	gastrointestinal disorders	1.29 (1.21 - 1.39)
	vomiting	716	gastrointestinal disorders	1.87 (1.74 - 2.02)
	hypotension	660	vascular disorders	4 (3.71 - 4.32)
	dehydration	656	metabolism and nutrition disorders	5.92 (5.48 - 6.4)
	pyrexia	631	general disorders and administration site conditions	2.16 (2 - 2.34)
	dermatitis acneiform	574	skin and subcutaneous tissue disorders	132.56 (121.49 - 144.62)
	neutropenia	559	blood and lymphatic system disorders	5.04 (4.64 - 5.48)
	mucosal inflammation	511	general disorders and administration site conditions	24.66 (22.58 - 26.93)
	pruritus	509	skin and subcutaneous tissue disorders	1.76 (1.61 - 1.92)
	anaemia	489	blood and lymphatic system disorders	3.04 (2.78 - 3.33)
	decreased appetite	461	metabolism and nutrition disorders	2.44 (2.23 - 2.68)
	dysphagia	446	gastrointestinal disorders	5.79 (5.27 - 6.36)
	hypersensitivity	439	immune system disorders	2.87 (2.61 - 3.15)
	white blood cell count decreased	434	investigations	4.76 (4.33 - 5.23)
	weight decreased	433	investigations	1.84 (1.67 - 2.02)
**Panitumumab**	rash	1204	skin and subcutaneous tissue disorders	5.97 (5.63 - 6.32)
	diarrhoea	893	gastrointestinal disorders	2.88 (2.69 - 3.08)
	death	671	general disorders and administration site conditions	1.63 (1.51 - 1.76)
	dermatitis acneiform	651	skin and subcutaneous tissue disorders	258.74 (238.16 - 281.1)
	hypomagnesaemi	524	metabolism and nutrition	83.35 (76.3 - 91.04)

a		disorders	
neutropenia	486	blood and lymphatic system disorders	7.38 (6.74 - 8.07)
nausea	440	gastrointestinal disorders	1.13 (1.03 - 1.24)
skin toxicity	437	skin and subcutaneous tissue disorders	198.52 (179.73 - 219.27)
vomiting	386	gastrointestinal disorders	1.69 (1.53 - 1.87)
stomatitis	382	gastrointestinal disorders	12.92 (11.68 - 14.3)
paronychia	366	infections and infestations	197.47 (177.17 - 220.1)
dry skin	353	skin and subcutaneous tissue disorders	5.79 (5.21 - 6.43)
decreased appetite	344	metabolism and nutrition disorders	3.06 (2.75 - 3.4)
neuropathy peripheral	316	nervous system disorders	7.1 (6.35 - 7.93)
pyrexia	277	general disorders and administration site conditions	1.58 (1.41 - 1.78)
anaemia	267	blood and lymphatic system disorders	2.78 (2.46 - 3.14)
thrombocytopenia	260	blood and lymphatic system disorders	4.76 (4.21 - 5.38)
pruritus	249	skin and subcutaneous tissue disorders	1.44 (1.27 - 1.63)
interstitial lung disease	239	respiratory, thoracic and mediastinal disorders	10.33 (9.09 - 11.74)
asthenia	237	general disorders and administration site conditions	1.26 (1.11 - 1.43)

Abbreviations: AEs, adverse events; PTs, preferred terms; ROR, reported odds ratio; SOC, systemic organ classes.

Supplementary Table 4. The top twenty AEs with the strongest signal intensity for cetuximab and panitumumab.

Drug	PT	Frequency	SOC	ROR (95% Cl)
**Cetuximab**	dermatitis acneiform	574	skin and subcutaneous tissue disorders	132.56 (121.49 - 144.62)
	trichomegaly	9	eye disorders	131.59 (65.84 - 263.01)
	pneumonia serratia	3	infections and infestations	91.48 (28.13 - 297.44)
	laryngeal necrosis	3	respiratory, thoracic and mediastinal disorders	84.25 (26.01 - 272.96)
	pharyngeal inflammation	77	respiratory, thoracic and mediastinal disorders	83.46 (66.18 - 105.27)
	mucosal infection	13	infections and infestations	67.69 (38.64 - 118.58)
	nail bed inflammation	16	skin and subcutaneous tissue disorders	65.95 (39.8 - 109.27)
	femoral artery embolism	3	vascular disorders	57.17 (17.9 - 182.66)
	rash follicular	7	skin and subcutaneous tissue disorders	56.6 (26.46 - 121.06)
	granulocyte count decreased	57	investigations	52.54 (40.27 - 68.56)
	paronychia	149	infections and infestations	44.66 (37.9 - 52.62)
	portal venous gas	11	gastrointestinal disorders	38.75 (21.23 - 70.72)
	skin toxicity	139	skin and subcutaneous tissue disorders	34.66 (29.27 - 41.05)
	pharyngeal stenosis	3	respiratory, thoracic and mediastinal disorders	34.43 (10.9 - 108.7)
	hypomagnesaemia	369	metabolism and nutrition disorders	34.22 (30.84 - 37.97)
	tracheostomy infection	3	infections and infestations	31.7 (10.05 - 99.95)
	nail bed tenderness	3	skin and subcutaneous tissue disorders	31.08 (9.86 - 97.97)
	cutaneous symptom	15	skin and subcutaneous tissue disorders	29.38 (17.59 - 49.08)
	thrombophlebitis migrans	6	vascular disorders	26.91 (11.97 - 60.5)
	mucosal inflammation	511	general disorders and administration site conditions	24.66 (22.58 - 26.93)
**Panitumumab**	trichomegaly	22	eye disorders	655.96 (402.4 -

			1069.28)
dermatitis acneiform	651	skin and subcutaneous tissue disorders	258.74 (238.16 - 281.1)
skin toxicity	437	skin and subcutaneous tissue disorders	198.52 (179.73 - 219.27)
paronychia	366	infections and infestations	197.47 (177.17 - 220.1)
xeroderma	14	skin and subcutaneous tissue disorders	121.55 (70.73 - 208.88)
adjusted calcium decreased	6	investigations	115.36 (50.52 - 263.4)
eyelash changes	7	eye disorders	94.82 (44.33 - 202.81)
nail bed inflammation	13	skin and subcutaneous tissue disorders	88.74 (50.84 - 154.9)
xerosis	48	general disorders and administration site conditions	87.43 (65.42 - 116.84)
hypomagnesaemia	524	metabolism and nutrition disorders	83.35 (76.3 - 91.04)
ectropion	13	eye disorders	81.29 (46.62 - 141.74)
mucocutaneous rash	5	skin and subcutaneous tissue disorders	77.07 (31.48 - 188.67)
dermatophytosis of nail	4	infections and infestations	74.5 (27.4 - 202.56)
acne pustular	26	infections and infestations	65.34 (44.17 - 96.65)
stoma site ulcer	5	injury, poisoning and procedural complications	64.78 (26.54 - 158.12)
growth of eyelashes	24	eye disorders	59.22 (39.43 - 88.96)
dermatitis infected	13	infections and infestations	53.57 (30.85 - 93.02)
eyelash thickening	3	eye disorders	50.13 (15.91 - 157.9)
rash pustular	165	infections and infestations	44.39 (38.02 - 51.82)
urinoma	3	renal and urinary disorders	41.26 (13.13 - 129.61)

Abbreviations: AEs, adverse events; PTs, preferred terms; ROR, reported odds ratio; SOC, systemic organ classes.

Supplementary Table 5. Signal strength of reports of cetuximab and panitumumab at the PT level in the FAERS database.

	Cetuximab			Panitumumab		
**SOC system**	**PTs**	**N**	**ROR (95% CI)**	**PTs**	**N**	**ROR (95% CI)**
**BLOOD AND LYMPHATIC SYSTEM DISORDERS**	neutropenia	559	5.04 (4.64 - 5.48)	neutropenia	486	7.38 (6.74 - 8.07)
	anaemia	489	3.04 (2.78 - 3.33)	anaemia	267	2.78 (2.46 - 3.14)
	thrombocytopenia	359	3.92 (3.53 - 4.35)	thrombocytopenia	260	4.76 (4.21 - 5.38)
	febrile neutropenia	330	6.22 (5.58 - 6.93)	febrile neutropenia	230	7.26 (6.37 - 8.26)
	leukopenia	277	6.73 (5.98 - 7.58)	leukopenia	115	4.66 (3.88 - 5.59)
	myelosuppression	252	15.23 (13.45 - 17.26)	bone marrow failure	41	3.82 (2.81 - 5.19)
	bone marrow failure	138	7.73 (6.53 - 9.14)	pancytopenia	38	1.4 (1.02 - 1.93)
	pancytopenia	81	1.79 (1.44 - 2.22)	myelosuppression	27	2.69 (1.84 - 3.92)
	lymphopenia	58	5 (3.87 - 6.48)	haematotoxicity	24	5.82 (3.89 - 8.68)
	disseminated intravascular coagulation	53	4.39 (3.35 - 5.75)	disseminated intravascular coagulation	22	3.04 (2 - 4.62)
	haematotoxicity	23	3.33 (2.21 - 5.01)	febrile bone marrow aplasia	21	10.62 (6.91 - 16.31)
	febrile bone marrow aplasia	22	6.64 (4.37 - 10.1)	lymphopenia	16	2.3 (1.41 - 3.76)
	granulocytopenia	19	3.76 (2.4 - 5.91)	granulocytopenia	8	2.65 (1.32 - 5.3)
	white blood cell disorder	7	3.05 (1.45 - 6.41)	lymphadenopathy mediastinal	5	5.35 (2.22 - 12.86)
	microcytic anaemia	5	2.83 (1.17 - 6.8)	thrombotic thrombocytopenic purpura	5	2.78 (1.16 - 6.7)
	/	/	/	thrombocytopenic purpura	3	4.22 (1.36 - 13.11)
**CARDIAC DISORDERS**	tachycardia	188	2.56 (2.21 - 2.95)	cardiopulmonary failure	6	2.98 (1.34 - 6.63)
	cardio-respiratory arrest	167	4.69 (4.03 - 5.46)	/	/	/
	cardiac arrest	139	2 (1.69 - 2.37)	/	/	/

	atrial fibrillation	124	1.52 (1.27 - 1.81)	/	/	/
	acute myocardial infarction	52	2.03 (1.55 - 2.67)	/	/	/
	sinus tachycardia	38	3.2 (2.32 - 4.39)	/	/	/
	angina pectoris	37	1.48 (1.07 - 2.04)	/	/	/
	myocardial ischaemia	32	3.13 (2.21 - 4.43)	/	/	/
	ventricular tachycardia	28	2.02 (1.4 - 2.93)	/	/	/
	supraventricular tachycardia	18	2.22 (1.4 - 3.53)	/	/	/
	acute coronary syndrome	16	2.21 (1.35 - 3.61)	/	/	/
	atrial flutter	15	2.19 (1.32 - 3.63)	/	/	/
	stress cardiomyopathy	13	2.94 (1.71 - 5.07)	/	/	/
	arrhythmia supraventricular	6	6.24 (2.79 - 13.91)	/	/	/
	diastolic dysfunction	6	2.32 (1.04 - 5.18)	/	/	/
	prinzmetal angina	5	6 (2.49 - 14.46)	/	/	/
	cardiovascular insufficiency	4	2.77 (1.04 - 7.4)	/	/	/
	left atrial dilatation	4	3.61 (1.35 - 9.64)	/	/	/
	mitral valve calcification	3	5.65 (1.82 - 17.56)	/	/	/
	nodal arrhythmia	3	3.98 (1.28 - 12.36)	/	/	/
**CONGENITAL, FAMILIAL AND GENETIC DISORDERS**	k-ras gene mutation	3	52.49 (16.47 - 167.28)	gene mutation	34	35.5 (25.28 - 49.86)
	/	/	/	k-ras gene mutation	6	184.97 (79.8 - 428.72)
	/	/	/	braf gene mutation	5	120.81 (48.84 - 298.85)
	/	/	/	acquired gene mutation	4	6.38 (2.39 - 17.03)

	/	/	/	phimosis	3	8.61 (2.77 - 26.77)
ENDOCRINE DISORDERS	hypercalcaemia of malignancy	3	14.82 (4.74 - 46.32)	/	/	/
EYE DISORDERS	blepharitis	25	8.18 (5.52 - 12.12)	eye irritation	38	1.56 (1.13 - 2.14)
	ulcerative keratitis	17	7.67 (4.76 - 12.37)	ocular hyperaemia	34	1.58 (1.13 - 2.21)
	periorbital oedema	11	2.59 (1.43 - 4.67)	growth of eyelashes	24	59.22 (39.43 - 88.96)
	growth of eyelashes	10	14.44 (7.74 - 26.96)	trichomegaly	22	655.96 (402.4 - 1069.28)
	ocular toxicity	9	13.25 (6.87 - 25.57)	blepharitis	21	11.5 (7.49 - 17.66)
	trichomegaly	9	131.59 (65.84 - 263.01)	keratitis	18	12.54 (7.89 - 19.93)
	keratitis	9	3.73 (1.94 - 7.17)	ocular toxicity	13	32.25 (18.63 - 55.81)
	serous retinal detachment	8	14.72 (7.33 - 29.58)	eye discharge	13	2.78 (1.61 - 4.79)
	eyelid margin crusting	5	2.51 (1.04 - 6.03)	ectropion	13	81.29 (46.62 - 141.74)
	eyelid disorder	5	2.61 (1.08 - 6.27)	eye inflammation	9	2.04 (1.06 - 3.91)
	corneal perforation	4	7.93 (2.97 - 21.22)	ulcerative keratitis	8	6.03 (3.01 - 12.06)
	corneal erosion	3	6.74 (2.17 - 20.97)	eyelid disorder	7	6.12 (2.91 - 12.85)
	trichiasis	3	11.48 (3.68 - 35.8)	eyelash changes	7	94.82 (44.33 - 202.81)
	/	/	/	xerophthalmia	5	18.98 (7.86 - 45.82)
	/	/	/	conjunctival hyperaemia	5	2.88 (1.2 - 6.93)
	/	/	/	punctate keratitis	3	7.2 (2.32 - 22.37)
	/	/	/	corneal perforation	3	9.95 (3.2 - 30.95)
	/	/	/	conjunctival disorder	3	15.59 (5 - 48.59)
	/	/	/	eyelash thickening	3	50.13 (15.91 - 157.9)
	/	/	/	eye allergy	3	3.97 (1.28 - 12.33)

	diarrhoea	895	1.7 (1.59 - 1.82)	diarrhoea	893	2.88 (2.69 - 3.08)
	nausea	843	1.29 (1.21 - 1.39)	nausea	440	1.13 (1.03 - 1.24)
	vomiting	716	1.87 (1.74 - 2.02)	vomiting	386	1.69 (1.53 - 1.87)
	dysphagia	446	5.79 (5.27 - 6.36)	stomatitis	382	12.92 (11.68 - 14.3)
	stomatitis	398	8 (7.25 - 8.83)	abdominal pain	167	1.44 (1.24 - 1.68)
	abdominal pain	272	1.4 (1.24 - 1.58)	intestinal obstruction	65	3.65 (2.86 - 4.65)
	intestinal obstruction	104	3.49 (2.88 - 4.23)	ileus	57	9.75 (7.51 - 12.65)
	dry mouth	103	1.55 (1.28 - 1.88)	ascites	55	3.73 (2.86 - 4.86)
	ileus	84	8.6 (6.93 - 10.66)	colitis	34	1.92 (1.37 - 2.69)
	oral pain	83	4.33 (3.49 - 5.37)	rectal haemorrhage	34	1.55 (1.1 - 2.17)
**GASTROINTESTINAL DISORDERS**	oesophagitis	81	9.12 (7.33 - 11.36)	mouth ulceration	25	2.41 (1.63 - 3.57)
	ascites	64	2.59 (2.02 - 3.31)	small intestinal obstruction	24	3.98 (2.67 - 5.94)
	colitis	56	1.89 (1.45 - 2.46)	intestinal perforation	24	4.36 (2.92 - 6.51)
	gastrointestinal toxicity	46	13.65 (10.21 - 18.26)	gastrointestinal toxicity	20	9.87 (6.36 - 15.32)
	haematemesis	42	1.95 (1.44 - 2.65)	aphthous ulcer	20	5.15 (3.32 - 7.99)
	pneumatosis intestinalis	42	18.89 (13.92 - 25.63)	subileus	18	16.66 (10.48 - 26.51)
	swollen tongue	42	1.61 (1.19 - 2.17)	enterocolitis	18	6.86 (4.32 - 10.9)
	odynophagia	39	8.27 (6.03 - 11.33)	cheilitis	16	5.89 (3.6 - 9.62)
	small intestinal obstruction	38	3.77 (2.74 - 5.18)	oesophagitis	16	3 (1.83 - 4.89)
	intestinal perforation	32	3.47 (2.45 - 4.91)	large intestine perforation	16	4.65 (2.85 - 7.6)
	melaena	31	1.66 (1.16 - 2.35)	gastrointestinal perforation	15	7.32 (4.41 - 12.15)
	mouth ulceration	30	1.73 (1.21 - 2.47)	duodenal ulcer	15	4.27 (2.57 - 7.09)

enteritis	30	5.95 (4.16 - 8.52)	oral disorder	15	4.09 (2.46 - 6.78)
large intestine perforation	27	4.69 (3.22 - 6.85)	glossitis	15	8.36 (5.03 - 13.88)
cheilitis	24	5.28 (3.54 - 7.89)	proctalgia	13	3.94 (2.28 - 6.78)
gastrointestinal perforation	22	6.42 (4.22 - 9.76)	enteritis	12	3.97 (2.26 - 7)
gastrointestinal necrosis	21	8.68 (5.65 - 13.34)	pneumatosis intestinalis	10	7.43 (3.99 - 13.83)
mouth haemorrhage	20	3.31 (2.14 - 5.14)	lip dry	8	2.12 (1.06 - 4.23)
duodenal ulcer haemorrhage	19	7.74 (4.93 - 12.15)	intestinal ischaemia	7	2.61 (1.24 - 5.48)
intestinal ischaemia	18	4.02 (2.53 - 6.38)	anal haemorrhage	7	5.03 (2.39 - 10.56)
chapped lips	16	4.47 (2.74 - 7.31)	anorectal discomfort	7	2.57 (1.22 - 5.4)
duodenal ulcer	16	2.72 (1.66 - 4.44)	malignant ascites	6	15.8 (7.07 - 35.3)
subileus	15	8.27 (4.98 - 13.75)	large intestinal obstruction	6	5.71 (2.56 - 12.73)
enterocolitis	14	3.18 (1.88 - 5.38)	angular cheilitis	6	19.09 (8.54 - 42.68)
tongue disorder	14	2.2 (1.31 - 3.73)	rectal perforation	6	22.17 (9.91 - 49.59)
proctalgia	14	2.53 (1.5 - 4.28)	tongue discolouration	6	2.6 (1.17 - 5.8)
colitis ischaemic	13	2.38 (1.38 - 4.1)	tongue discomfort	5	2.49 (1.04 - 5.99)
lip dry	12	1.9 (1.08 - 3.34)	oesophageal ulcer	5	2.58 (1.07 - 6.21)
aphthous ulcer	12	1.84 (1.05 - 3.25)	large intestinal stenosis	5	5.52 (2.29 - 13.28)
ileus paralytic	11	3.04 (1.68 - 5.49)	colonic fistula	4	13.89 (5.19 - 37.14)
portal venous gas	11	38.75 (21.23 - 70.72)	oesophageal varices haemorrhage	4	3.68 (1.38 - 9.82)
oesophageal pain	11	3.26 (1.8 - 5.89)	mechanical ileus	4	15.35 (5.74 - 41.07)
anal fissure	10	2.07 (1.11 - 3.86)	anorectal disorder	4	3.93 (1.47 - 10.48)
diverticular perforation	8	4.5 (2.25 - 9.01)	dyschezia	4	2.88 (1.08 - 7.67)

small intestinal perforation	8	5.25 (2.62 - 10.52)	enterocutaneous fistula	4	8.89 (3.33 - 23.76)
tongue discolouration	8	2.07 (1.04 - 4.15)	proctitis	4	2.84 (1.06 - 7.56)
oesophageal stenosis	8	3.42 (1.71 - 6.85)	anal ulcer	3	7.82 (2.52 - 24.3)
oral mucosal erythema	8	6.21 (3.1 - 12.44)	small intestinal perforation	3	3.29 (1.06 - 10.21)
duodenal perforation	7	9.12 (4.33 - 19.2)	enterocolitis haemorrhagic	3	3.88 (1.25 - 12.05)
glossitis	7	2.32 (1.11 - 4.87)	oral mucosal eruption	3	3.99 (1.28 - 12.38)
tongue haemorrhage	7	7.6 (3.61 - 15.99)	tongue dry	3	4.4 (1.42 - 13.65)
oesophageal ulcer	7	2.16 (1.03 - 4.54)	small intestine ulcer	3	9.51 (3.06 - 29.58)
tongue ulceration	7	2.42 (1.15 - 5.09)	ileal perforation	3	9.04 (2.91 - 28.13)
lip haemorrhage	6	5.62 (2.52 - 12.53)	enterovesical fistula	3	8.01 (2.58 - 24.89)
lip pain	6	2.49 (1.12 - 5.55)	anal inflammation	3	8.79 (2.83 - 27.34)
rectal perforation	6	13.23 (5.91 - 29.6)	duodenal perforation	3	6.52 (2.1 - 20.25)
enterocutaneous fistula	6	7.98 (3.58 - 17.83)	/	/	/
oesophageal obstruction	6	8.64 (3.87 - 19.3)	/	/	/
large intestinal ulcer	6	2.67 (1.2 - 5.96)	/	/	/
acute abdomen	5	3.97 (1.65 - 9.56)	/	/	/
oesophageal haemorrhage	5	4.43 (1.84 - 10.67)	/	/	/
necrotising oesophagitis	5	15.93 (6.59 - 38.52)	/	/	/
gastrointestinal ulcer	5	2.63 (1.09 - 6.32)	/	/	/
pneumoperitoneum	5	3.34 (1.39 - 8.02)	/	/	/
intestinal dilatation	5	4.66 (1.94 - 11.23)	/	/	/
saliva altered	4	5.31 (1.99 - 14.18)	/	/	/

	anal ulcer	4	6.23 (2.33 - 16.65)	/	/	/
	oesophageal perforation	4	5.7 (2.13 - 15.23)	/	/	/
	salivary gland disorder	4	9.72 (3.63 - 26.03)	/	/	/
	oesophageal fistula	4	15.3 (5.7 - 41.06)	/	/	/
	rectal tenesmus	3	3.16 (1.02 - 9.82)	/	/	/
	anal inflammation	3	5.25 (1.69 - 16.32)	/	/	/
	gastric fistula	3	8.8 (2.82 - 27.4)	/	/	/
	ileal perforation	3	5.4 (1.74 - 16.79)	/	/	/
	colonic fistula	3	6.2 (1.99 - 19.3)	/	/	/
	oral cavity fistula	3	6.81 (2.19 - 21.2)	/	/	/
**GENERAL DISORDERS AND ADMINISTRATION SITE CONDITIONS**	pyrexia	631	2.16 (2 - 2.34)	death	671	1.63 (1.51 - 1.76)
	mucosal inflammation	511	24.66 (22.58 - 26.93)	disease progression	449	8.01 (7.29 - 8.79)
	asthenia	414	1.31 (1.19 - 1.45)	pyrexia	277	1.58 (1.41 - 1.78)
	chills	359	3.63 (3.27 - 4.03)	asthenia	237	1.26 (1.11 - 1.43)
	general physical health deterioration	214	2.38 (2.08 - 2.72)	therapy partial responder	169	32.83 (28.18 - 38.24)
	chest pain	209	1.33 (1.16 - 1.52)	mucosal inflammation	154	12.19 (10.4 - 14.29)
	chest discomfort	162	1.94 (1.67 - 2.27)	general physical health deterioration	131	2.44 (2.05 - 2.9)
	adverse event	129	1.7 (1.43 - 2.02)	adverse event	67	1.48 (1.16 - 1.88)
	disease progression	120	1.26 (1.05 - 1.5)	xerosis	48	87.43 (65.42 - 116.84)
	feeling hot	68	1.31 (1.03 - 1.66)	inflammation	39	1.57 (1.14 - 2.15)
	extravasation	63	18.53 (14.44 - 23.77)	therapy non-responder	35	1.77 (1.27 - 2.46)
	feeling cold	38	1.61 (1.17 - 2.21)	drug resistance	24	1.96 (1.31 - 2.92)

	face oedema	32	2.24 (1.58 - 3.16)	face oedema	16	1.87 (1.15 - 3.05)
	performance status decreased	28	7 (4.82 - 10.15)	performance status decreased	14	5.84 (3.46 - 9.87)
	sudden death	25	2.68 (1.81 - 3.98)	secretion discharge	11	1.87 (1.04 - 3.38)
	hyperthermia	21	3.32 (2.16 - 5.09)	facial pain	9	2.03 (1.06 - 3.9)
	infusion site extravasation	20	3.56 (2.29 - 5.52)	infusion site extravasation	8	2.38 (1.19 - 4.76)
	necrosis	19	4.03 (2.57 - 6.32)	necrosis	7	2.48 (1.18 - 5.21)
	temperature intolerance	15	1.8 (1.08 - 2.98)	mucosal toxicity	5	39.38 (16.23 - 95.54)
	localised oedema	13	3.3 (1.91 - 5.69)	mucosal disorder	4	8.97 (3.36 - 23.97)
	hyperpyrexia	13	4.32 (2.5 - 7.44)	administration site extravasation	3	6.76 (2.17 - 20.99)
	infusion site reaction	12	6.35 (3.6 - 11.2)	device related thrombosis	3	6.72 (2.16 - 20.89)
	foaming at mouth	10	5.95 (3.2 - 11.08)	/	/	/
	systemic inflammatory response syndrome	7	2.14 (1.02 - 4.48)	/	/	/
	physical deconditioning	7	4.23 (2.01 - 8.88)	/	/	/
	catheter site pain	6	3.06 (1.37 - 6.81)	/	/	/
	pneumatosis	5	13.75 (5.69 - 33.23)	/	/	/
	xerosis	4	4.16 (1.56 - 11.1)	/	/	/
	catheter site discharge	4	4.63 (1.73 - 12.35)	/	/	/
	pelvic mass	4	5.9 (2.21 - 15.78)	/	/	/
	mucosal ulceration	3	3.57 (1.15 - 11.1)	/	/	/
	glassy eyes	3	5.25 (1.69 - 16.32)	/	/	/
	mucosal toxicity	3	13.98 (4.48 - 43.67)	/	/	/
**HEPATOBILIARY**	hepatic function	73	2.44 (1.94 -	hepatic failure	53	3.51 (2.68 -

DISORDERS	abnormal		3.07)			4.59)
	hepatic failure	42	1.66 (1.22 - 2.24)	hepatic function abnormal	39	2.18 (1.59 - 2.98)
	jaundice	36	1.56 (1.12 - 2.16)	jaundice	36	2.61 (1.88 - 3.62)
	hyperbilirubinaemia	31	3.72 (2.61 - 5.29)	hepatotoxicity	25	2.34 (1.58 - 3.47)
	cholangitis	20	4.19 (2.7 - 6.5)	cholangitis	19	6.67 (4.25 - 10.47)
	portal vein thrombosis	10	3.79 (2.04 - 7.06)	hepatic lesion	17	8.03 (4.99 - 12.94)
	jaundice cholestatic	7	2.34 (1.11 - 4.91)	hyperbilirubinaemia	14	2.81 (1.66 - 4.74)
	biliary dilatation	7	5.69 (2.71 - 11.97)	venoocclusive liver disease	12	5.04 (2.86 - 8.89)
	hepatorenal syndrome	4	2.74 (1.03 - 7.31)	portal vein thrombosis	12	7.63 (4.33 - 13.46)
	hepatic vein thrombosis	3	10.93 (3.5 - 34.08)	hypertransaminasaemia	9	3.28 (1.7 - 6.3)
	cholangitis acute	3	5.14 (1.65 - 15.98)	jaundice cholestatic	7	3.92 (1.87 - 8.23)
	/	/	/	bile duct stone	5	3.17 (1.32 - 7.61)
	/	/	/	hepatic fibrosis	5	3.01 (1.25 - 7.24)
	/	/	/	bile duct stenosis	3	5.08 (1.64 - 15.79)
	/	/	/	biloma	3	49.66 (15.77 - 156.42)
	/	/	/	biliary obstruction	3	6.5 (2.09 - 20.2)
	hypersensitivity	439	2.87 (2.61 - 3.15)	decreased immune responsiveness	27	5.66 (3.88 - 8.25)
	anaphylactic reaction	407	9.56 (8.67 - 10.54)	/	/	/
**IMMUNE SYSTEM DISORDERS**	anaphylactic shock	134	6.63 (5.6 - 7.86)	/	/	/
	immunodeficiency	68	6.03 (4.75 - 7.65)	/	/	/
	anaphylactoid reaction	23	6.74 (4.47 - 10.15)	/	/	/
	cytokine release syndrome	22	1.92 (1.26 - 2.91)	/	/	/

	infusion related hypersensitivity reaction	8	23.14 (11.49 - 46.62)	/	/	/
	cytokine storm	3	5.68 (1.83 - 17.65)	/	/	/
	pneumonia	400	1.51 (1.37 - 1.66)	paronychia	366	197.47 (177.17 - 220.1)
	sepsis	263	2.83 (2.5 - 3.19)	rash pustular	165	44.39 (38.02 - 51.82)
	paronychia	149	44.66 (37.9 - 52.62)	urinary tract infection	121	1.43 (1.19 - 1.7)
	pneumonia aspiration	137	6.69 (5.65 - 7.91)	sepsis	111	1.99 (1.65 - 2.4)
	septic shock	122	3.52 (2.95 - 4.21)	infection	97	1.38 (1.13 - 1.69)
	rash pustular	97	15.37 (12.57 - 18.78)	conjunctivitis	84	9.66 (7.8 - 11.98)
	staphylococcal infection	86	3.04 (2.46 - 3.76)	folliculitis	78	18.23 (14.58 - 22.79)
	cellulitis	80	1.84 (1.48 - 2.3)	device related infection	55	6.9 (5.29 - 8.99)
	conjunctivitis	69	4.73 (3.73 - 5.99)	septic shock	32	1.54 (1.09 - 2.18)
**INFECTIONS AND INFESTATIONS**	device related infection	53	3.97 (3.03 - 5.19)	skin infection	26	4.72 (3.21 - 6.93)
	skin infection	36	3.9 (2.81 - 5.41)	acne pustular	26	65.34 (44.17 - 96.65)
	peritonitis	34	1.94 (1.39 - 2.72)	neutropenic sepsis	24	6.57 (4.4 - 9.81)
	folliculitis	33	4.57 (3.24 - 6.43)	pustule	20	21.07 (13.56 - 32.75)
	meningitis aseptic	33	9.26 (6.57 - 13.05)	abscess	17	2.09 (1.3 - 3.36)
	bacteraemia	31	3.3 (2.32 - 4.69)	gastroenteritis	17	2.33 (1.45 - 3.75)
	pneumonia bacterial	24	3.33 (2.23 - 4.98)	pyelonephritis	15	3.5 (2.11 - 5.8)
	staphylococcal sepsis	22	5.14 (3.38 - 7.81)	subcutaneous abscess	15	6.09 (3.67 - 10.11)
	oral candidiasis	19	1.8 (1.15 - 2.83)	abdominal abscess	15	6.96 (4.19 - 11.55)
	neutropenic sepsis	18	2.94 (1.85 - 4.67)	dermatitis infected	13	53.57 (30.85 - 93.02)
	wound infection	17	2.11 (1.31 -	furuncle	13	3.2 (1.85 -

		3.39)			5.51)
nail infection	15	12.44 (7.48 - 20.7)	necrotising fasciitis	12	7.14 (4.05 - 12.58)
mucosal infection	13	67.69 (38.64 - 118.58)	anal abscess	11	4.13 (2.29 - 7.47)
superinfection	13	10.34 (5.99 - 17.86)	pneumocystis jirovecii pneumonia	10	2.16 (1.16 - 4.01)
staphylococcal bacteraemia	12	3.51 (1.99 - 6.19)	pelvic abscess	10	17.64 (9.46 - 32.88)
escherichia infection	12	1.83 (1.04 - 3.23)	staphylococcal sepsis	10	3.9 (2.1 - 7.26)
clostridial infection	12	3.95 (2.24 - 6.96)	erysipelas	9	3.5 (1.82 - 6.73)
pneumonia staphylococcal	11	6.61 (3.66 - 11.97)	staphylococcal skin infection	9	14.39 (7.47 - 27.74)
abdominal abscess	10	2.76 (1.49 - 5.14)	superinfection	7	9.29 (4.42 - 19.52)
acne pustular	9	13.18 (6.83 - 25.43)	herpes virus infection	7	2.42 (1.15 - 5.08)
endocarditis	9	2.14 (1.11 - 4.11)	nail infection	7	9.67 (4.6 - 20.32)
lung abscess	8	4.7 (2.35 - 9.42)	vascular device infection	7	3.21 (1.53 - 6.75)
enterocolitis infectious	8	9.74 (4.85 - 19.53)	catheter site infection	6	3.27 (1.47 - 7.29)
intervertebral discitis	7	4.56 (2.17 - 9.58)	conjunctivitis bacterial	6	32.61 (14.54 - 73.12)
soft tissue infection	7	4.64 (2.21 - 9.75)	pneumonia staphylococcal	6	6.03 (2.7 - 13.43)
neutropenic infection	7	8.33 (3.96 - 17.52)	impetigo	6	8.32 (3.73 - 18.56)
oesophageal candidiasis	7	2.21 (1.05 - 4.65)	superinfection bacterial	6	17 (7.61 - 37.99)
catheter site infection	7	2.28 (1.09 - 4.78)	liver abscess	6	3.81 (1.71 - 8.49)
fungaemia	6	4.83 (2.17 - 10.78)	staphylococcal bacteraemia	5	2.45 (1.02 - 5.88)
pneumonia necrotising	6	13.34 (5.96 - 29.85)	infected skin ulcer	4	4.06 (1.52 - 10.82)
pneumonia klebsiella	6	3.98 (1.79 - 8.88)	device related sepsis	4	3.78 (1.42 - 10.07)
post procedural sepsis	6	11.73 (5.25 - 26.22)	dysentery	4	4.31 (1.62 - 11.51)
purulence	5	2.88 (1.2 -	abdominal infection	4	3.19 (1.2 -

		6.92)			8.51)
impetigo	5	4.14 (1.72 - 9.96)	dermatophytosis of nail	4	74.5 (27.4 - 202.56)
pyoderma	5	11.78 (4.88 - 28.44)	abdominal sepsis	4	9.77 (3.66 - 26.1)
wound infection staphylococcal	5	3.21 (1.33 - 7.72)	nail bed infection	4	33.89 (12.6 - 91.15)
device related sepsis	5	2.82 (1.17 - 6.78)	purulence	4	3.85 (1.44 - 10.27)
endocarditis staphylococcal	5	12.13 (5.02 - 29.28)	febrile infection	3	5.35 (1.72 - 16.61)
dysentery	5	3.22 (1.34 - 7.75)	biliary tract infection	3	10.12 (3.25 - 31.48)
postoperative abscess	5	9.03 (3.74 - 21.77)	klebsiella sepsis	3	5.4 (1.74 - 16.78)
clostridium colitis	5	4.6 (1.91 - 11.06)	enterocolitis infectious	3	6.08 (1.96 - 18.89)
enteritis infectious	5	3.43 (1.43 - 8.26)	postoperative abscess	3	9.04 (2.91 - 28.13)
staphylococcal skin infection	4	3.8 (1.42 - 10.15)	hepatitis viral	3	7.38 (2.37 - 22.93)
tracheobronchitis	4	6.2 (2.32 - 16.58)	lymphangitis	3	7.32 (2.35 - 22.74)
pulmonary sepsis	4	3.37 (1.26 - 9.01)	pneumonia streptococcal	3	5.54 (1.78 - 17.19)
dermatitis infected	4	9.64 (3.6 - 25.79)	/	/	/
extradural abscess	4	6.19 (2.32 - 16.53)	/	/	/
biliary tract infection	4	8.07 (3.02 - 21.58)	/	/	/
sputum purulent	4	6.71 (2.51 - 17.94)	/	/	/
pseudomonal sepsis	4	2.8 (1.05 - 7.48)	/	/	/
meningitis bacterial	4	3.37 (1.26 - 8.99)	/	/	/
abdominal sepsis	4	5.83 (2.18 - 15.58)	/	/	/
tracheitis	3	4.72 (1.52 - 14.66)	/	/	/
alpha haemolytic streptococcal infection	3	4.01 (1.29 - 12.45)	/	/	/

	infected fistula	3	6.52 (2.1 - 20.29)	/	/	/
	endocarditis bacterial	3	4.61 (1.48 - 14.32)	/	/	/
	pyuria	3	3.54 (1.14 - 10.99)	/	/	/
	nail bed infection	3	15.1 (4.83 - 47.2)	/	/	/
	skin bacterial infection	3	4.76 (1.53 - 14.81)	/	/	/
	tracheostomy infection	3	31.7 (10.05 - 99.95)	/	/	/
	biliary sepsis	3	7.38 (2.37 - 22.96)	/	/	/
	pneumonia serratia	3	91.48 (28.13 - 297.44)	/	/	/
	febrile infection	3	3.19 (1.03 - 9.91)	/	/	/
	klebsiella sepsis	3	3.22 (1.04 - 10.01)	/	/	/
**INJURY, POISONING AND PROCEDURAL COMPLICATIONS**	off label use	1065	1.6 (1.5 - 1.7)	product storage error	121	3.44 (2.88 - 4.11)
	infusion related reaction	943	18.25 (17.1 - 19.47)	infusion related reaction	108	3.4 (2.81 - 4.1)
	radiation skin injury	176	211.77 (180.11 - 249)	circumstance or information capable of leading to medication error	98	6.43 (5.27 - 7.84)
	radiation mucositis	39	365.37 (253.97 - 525.63)	intercepted product administration error	23	6.7 (4.45 - 10.1)
	post procedural haemorrhage	25	2.51 (1.69 - 3.71)	spinal compression fracture	18	3.89 (2.45 - 6.18)
	radiation pneumonitis	20	11.22 (7.22 - 17.43)	post procedural complication	17	1.72 (1.07 - 2.77)
	skin wound	12	9.62 (5.45 - 16.99)	intercepted product preparation error	15	3.02 (1.82 - 5.01)
	radiation injury	12	13.7 (7.75 - 24.21)	occupational exposure to product	12	3.76 (2.13 - 6.63)
	osteoradionecrosis	11	55.91 (30.49 - 102.52)	skin laceration	12	2.27 (1.29 - 3.99)
	recall phenomenon	9	13.05 (6.76 - 25.19)	stoma site haemorrhage	10	13.09 (7.03 - 24.39)
	stoma site haemorrhage	8	6.24 (3.11 - 12.51)	sunburn	9	2.34 (1.22 - 4.5)

	tracheostomy malfunction	7	41.05 (19.29 - 87.34)	product preparation issue	9	3.81 (1.98 - 7.33)
	tracheal haemorrhage	5	16.99 (7.02 - 41.12)	skin injury	8	4.9 (2.45 - 9.82)
	jaw fracture	5	2.64 (1.1 - 6.34)	gastrointestinal stoma complication	7	8.77 (4.17 - 18.43)
	radiation dysphagia	5	156.95 (61.38 - 401.32)	stoma complication	7	12.78 (6.08 - 26.89)
	radiation oesophagitis	5	15.42 (6.38 - 37.29)	radiation skin injury	7	11.82 (5.62 - 24.85)
	gastrointestinal stoma complication	5	3.73 (1.55 - 8.99)	mouth injury	6	7.85 (3.52 - 17.5)
	endotracheal intubation complication	4	7.91 (2.96 - 21.14)	anastomotic leak	6	17.56 (7.86 - 39.24)
	arterial injury	4	6.33 (2.37 - 16.93)	stoma site ulcer	5	64.78 (26.54 - 158.12)
	injection related reaction	4	3.36 (1.26 - 8.98)	procedural haemorrhage	5	2.81 (1.17 - 6.76)
	procedural site reaction	4	8.99 (3.36 - 24.05)	post procedural bile leak	5	30 (12.39 - 72.61)
	gastroenteritis radiation	4	14.28 (5.32 - 38.29)	skin wound	5	6.68 (2.78 - 16.08)
	suture rupture	3	5.1 (1.64 - 15.85)	eschar	5	12.79 (5.31 - 30.83)
	radiation associated pain	3	18.3 (5.84 - 57.28)	stoma site inflammation	4	10.23 (3.83 - 27.34)
	gastrostomy tube site complication	3	19.06 (6.08 - 59.69)	burn oral cavity	4	14.08 (5.26 - 37.66)
	tracheal injury	3	25.61 (8.15 - 80.5)	eye injury	4	2.75 (1.03 - 7.33)
	/	/	/	stoma site pain	3	3.76 (1.21 - 11.67)
	/	/	/	subcutaneous haematoma	3	4.52 (1.46 - 14.04)
	/	/	/	nail injury	3	10.5 (3.37 - 32.65)
	/	/	/	postoperative adhesion	3	8.21 (2.64 - 25.54)
	/	/	/	gastrointestinal anastomotic leak	3	14.9 (4.78 - 46.42)
**INVESTIGATIONS**	white blood cell count decreased	434	4.76 (4.33 - 5.23)	neutrophil count decreased	194	9.77 (8.48 - 11.26)
	weight decreased	433	1.84 (1.67 -	platelet count	115	2.13 (1.78 -

		2.02)	decreased		2.56)
neutrophil count decreased	285	8.59 (7.64 - 9.65)	blood magnesium decreased	112	26.49 (21.98 - 31.94)
platelet count decreased	270	3 (2.66 - 3.38)	white blood cell count decreased	112	2.04 (1.69 - 2.46)
blood pressure decreased	260	4.68 (4.15 - 5.29)	carcinoembryonic antigen increased	53	56.73 (43.15 - 74.59)
oxygen saturation decreased	234	5.23 (4.6 - 5.95)	alanine aminotransferase increased	49	1.56 (1.18 - 2.07)
haemoglobin decreased	169	1.91 (1.65 - 2.23)	aspartate aminotransferase increased	49	1.81 (1.37 - 2.39)
aspartate aminotransferase increased	131	2.89 (2.44 - 3.44)	blood bilirubin increased	38	2.75 (2 - 3.79)
alanine aminotransferase increased	126	2.41 (2.02 - 2.87)	blood alkaline phosphatase increased	30	2.31 (1.61 - 3.3)
blood magnesium decreased	119	16.8 (14.01 - 20.13)	blood potassium decreased	26	1.71 (1.17 - 2.52)
blood creatinine increased	89	1.59 (1.29 - 1.96)	tumour marker increased	22	8.99 (5.91 - 13.66)
blood bilirubin increased	72	3.12 (2.48 - 3.93)	blood calcium decreased	19	3.26 (2.08 - 5.11)
blood alkaline phosphatase increased	68	3.13 (2.47 - 3.97)	blood albumin decreased	13	3.23 (1.87 - 5.56)
pulse absent	59	14.64 (11.32 - 18.93)	amylase increased	12	6.72 (3.81 - 11.85)
granulocyte count decreased	57	52.54 (40.27 - 68.56)	carbohydrate antigen 19-9 increased	9	37.6 (19.43 - 72.77)
blood potassium decreased	49	1.93 (1.46 - 2.55)	blood magnesium abnormal	6	22.73 (10.16 - 50.85)
blood sodium decreased	43	2.75 (2.04 - 3.71)	adjusted calcium decreased	6	115.36 (50.52 - 263.4)
body temperature increased	43	2.38 (1.76 - 3.21)	blood phosphorus decreased	5	2.75 (1.15 - 6.62)
lymphocyte count decreased	42	2.72 (2.01 - 3.68)	eastern cooperative oncology group performance status worsened	4	6.9 (2.58 - 18.41)
blood pressure immeasurable	38	29.99 (21.73 - 41.41)	staphylococcus test positive	4	3.7 (1.39 - 9.87)

haematocrit decreased	32	1.81 (1.28 - 2.56)	aspartate aminotransferase abnormal	4	5.93 (2.22 - 15.81)
blood urea increased	29	1.96 (1.36 - 2.82)	blood alkaline phosphatase abnormal	4	9.76 (3.65 - 26.07)
blood calcium decreased	26	2.66 (1.81 - 3.91)	blood alkaline phosphatase	3	32.51 (10.38 - 101.83)
carcinoembryonic antigen increased	25	15.69 (10.57 - 23.29)	blood bilirubin abnormal	3	4.93 (1.59 - 15.3)
blood lactate dehydrogenase increased	23	1.63 (1.08 - 2.45)	escherichia test positive	3	4.98 (1.6 - 15.47)
blood albumin decreased	19	2.82 (1.8 - 4.42)	/	/	/
blood culture positive	18	6.65 (4.18 - 10.57)	/	/	/
respiratory rate increased	17	2.25 (1.4 - 3.63)	/	/	/
staphylococcus test positive	13	7.22 (4.18 - 12.45)	/	/	/
culture urine positive	13	10.36 (6 - 17.9)	/	/	/
blood pressure systolic decreased	12	3.1 (1.76 - 5.46)	/	/	/
amylase increased	12	4.01 (2.28 - 7.07)	/	/	/
troponin increased	11	1.91 (1.06 - 3.45)	/	/	/
pulse abnormal	10	4.98 (2.67 - 9.27)	/	/	/
pulse pressure decreased	10	22.14 (11.84 - 41.42)	/	/	/
haemoglobin	8	51.13 (25.15 - 103.94)	/	/	/
blood magnesium abnormal	7	15.86 (7.52 - 33.46)	/	/	/
oxygen saturation immeasurable	7	98.31 (45.32 - 213.22)	/	/	/
sputum culture positive	7	9.27 (4.4 - 19.51)	/	/	/
bilirubin conjugated increased	6	3.05 (1.37 - 6.81)	/	/	/

	blood electrolytes decreased	6	5.42 (2.43 - 12.09)	/	/	/
	troponin i increased	6	4.37 (1.96 - 9.75)	/	/	/
	computerised tomogram abnormal	5	2.47 (1.03 - 5.94)	/	/	/
	blood electrolytes abnormal	4	3.83 (1.44 - 10.23)	/	/	/
	blood creatine phosphokinase mb increased	4	3.39 (1.27 - 9.06)	/	/	/
	neutrophil count	4	23.72 (8.8 - 63.88)	/	/	/
	laboratory test interference	3	4.33 (1.39 - 13.46)	/	/	/
	procalcitonin increased	3	3.45 (1.11 - 10.72)	/	/	/
	carbohydrate antigen 19-9 increased	3	7.38 (2.37 - 22.96)	/	/	/
	electrocardiogram change	3	3.69 (1.19 - 11.46)	/	/	/
	culture throat positive	3	62.78 (19.59 - 201.15)	/	/	/
**METABOLISM AND NUTRITION DISORDERS**	dehydration	656	5.92 (5.48 - 6.4)	hypomagnesaemia	524	83.35 (76.3 - 91.04)
	decreased appetite	461	2.44 (2.23 - 2.68)	decreased appetite	344	3.06 (2.75 - 3.4)
	hypomagnesaemia	369	34.22 (30.84 - 37.97)	dehydration	231	3.46 (3.04 - 3.94)
	hypokalaemia	198	5.24 (4.56 - 6.03)	hypokalaemia	131	5.81 (4.89 - 6.89)
	hyponatraemia	166	3.54 (3.04 - 4.12)	hypocalcaemia	113	12.28 (10.2 - 14.78)
	hypocalcaemia	128	8.3 (6.97 - 9.88)	hyponatraemia	43	1.53 (1.13 - 2.06)
	hyperkalaemia	82	2.87 (2.31 - 3.57)	hyperkalaemia	33	1.93 (1.37 - 2.72)
	hypophagia	80	3.89 (3.12 - 4.85)	electrolyte imbalance	31	5.62 (3.95 - 8)
	hypoalbuminaemia	59	9.27 (7.17 - 11.97)	hyperglycaemia	30	1.65 (1.15 - 2.36)
	malnutrition	57	6.38 (4.92 - 8.28)	hypoalbuminaemia	29	7.59 (5.27 - 10.94)
	electrolyte	48	5.2 (3.92 -	hypophagia	20	1.62 (1.05 -

	imbalance		6.91)			2.52)
	hyperglycaemia	46	1.51 (1.13 - 2.01)	malnutrition	16	2.99 (1.83 - 4.88)
	feeding disorder	36	2.42 (1.75 - 3.36)	hypophosphataemia	13	3.6 (2.09 - 6.2)
	hypophosphataemia	33	5.47 (3.88 - 7.7)	hyperammonaemia	9	3.73 (1.94 - 7.17)
	cachexia	31	7.07 (4.97 - 10.07)	tumour lysis syndrome	9	2.15 (1.12 - 4.14)
	hypernatraemia	21	5.14 (3.35 - 7.89)	tetany	8	8.71 (4.35 - 17.45)
	hypercalcaemia	21	2.04 (1.33 - 3.13)	cachexia	8	3.04 (1.52 - 6.08)
	failure to thrive	19	4.29 (2.73 - 6.73)	hypernatraemia	7	2.86 (1.36 - 6)
	hypovolaemia	19	3.9 (2.49 - 6.12)	hyperamylasaemia	6	21.72 (9.71 - 48.58)
	fluid intake reduced	10	2.52 (1.36 - 4.7)	failure to thrive	6	2.26 (1.02 - 5.04)
	tetany	8	5.2 (2.6 - 10.42)	calciphylaxis	5	5.28 (2.19 - 12.7)
	hypermagnesaemia	7	13.73 (6.52 - 28.95)	magnesium deficiency	4	18.67 (6.97 - 50.01)
	hypochloraemia	5	5.28 (2.19 - 12.72)	cell death	4	4.06 (1.52 - 10.83)
	hypercreatininaemia	4	9.77 (3.65 - 26.15)	hypoproteinaemia	3	3.32 (1.07 - 10.31)
	hyperamylasaemia	3	6.44 (2.07 - 20.04)	hypermagnesaemia	3	9.79 (3.15 - 30.44)
	magnesium deficiency	3	8.34 (2.68 - 25.97)	/	/	/
	electrolyte depletion	3	7.53 (2.42 - 23.45)	/	/	/
**MUSCULOSKELETAL AND CONNECTIVE TISSUE DISORDERS**	trismus	10	2.35 (1.26 - 4.36)	sarcopenia	3	19.72 (6.32 - 61.53)
**NEOPLASMS BENIGN, MALIGNANT AND UNSPECIFIED (INCL CYSTS AND POLYPS)**	malignant neoplasm progression	636	7.95 (7.35 - 8.6)	colorectal cancer metastatic	318	535.96 (472.66 - 607.73)
	metastases to lung	48	5.21 (3.92 - 6.92)	colon cancer	216	29.76 (26 - 34.06)
	metastases to liver	38	2.67 (1.94 - 3.67)	metastases to liver	195	23.31 (20.23 - 26.86)

metastases to central nervous system	30	3 (2.1 - 4.29)	colorectal cancer	146	5.54 (4.71 - 6.52)
neoplasm recurrence	30	10.7 (7.47 - 15.34)	malignant neoplasm progression	125	2.58 (2.17 - 3.08)
tumour haemorrhage	26	11.3 (7.68 - 16.64)	metastases to lung	87	15.92 (12.89 - 19.67)
oncologic complication	26	32.93 (22.29 - 48.66)	adenocarcinoma of colon	41	45.56 (33.41 - 62.12)
metastases to lymph nodes	20	3.77 (2.43 - 5.85)	neoplasm progression	36	1.93 (1.39 - 2.68)
tumour pain	17	15.21 (9.42 - 24.55)	metastases to central nervous system	28	4.69 (3.24 - 6.8)
metastasis	16	2.7 (1.65 - 4.41)	metastases to lymph nodes	27	8.54 (5.85 - 12.46)
colorectal cancer metastatic	13	10.08 (5.84 - 17.4)	colon cancer metastatic	24	30.76 (20.54 - 46.05)
metastases to spine	12	4.45 (2.52 - 7.84)	rectal cancer	23	15.17 (10.06 - 22.87)
cancer pain	12	5.74 (3.25 - 10.12)	metastases to peritoneum	22	19 (12.48 - 28.93)
lymphangiosis carcinomatosa	11	12.36 (6.82 - 22.39)	metastases to bone	21	2.7 (1.76 - 4.14)
recurrent cancer	9	2.8 (1.45 - 5.38)	metastasis	21	5.95 (3.87 - 9.13)
metastases to peritoneum	9	4.61 (2.39 - 8.87)	colorectal cancer recurrent	20	470.75 (287.62 - 770.5)
colon cancer metastatic	8	6.05 (3.02 - 12.12)	rectal cancer metastatic	17	104.85 (64.29 - 171.02)
non-small cell lung cancer	8	2.09 (1.04 - 4.18)	hepatic neoplasm	13	7.85 (4.55 - 13.54)
squamous cell carcinoma of head and neck	6	33.01 (14.65 - 74.39)	adenocarcinoma	11	9.54 (5.27 - 17.25)
tumour necrosis	5	5.64 (2.34 - 13.58)	metastases to ovary	10	66.98 (35.62 - 125.93)
tumour perforation	4	16.81 (6.26 - 45.13)	colon cancer recurrent	10	77.75 (41.28 - 146.46)
bone neoplasm	4	4.33 (1.62 - 11.57)	cancer pain	9	7.2 (3.74 - 13.86)
metastases to abdominal cavity	4	9.93 (3.71 - 26.57)	rectal adenocarcinoma	7	24.74 (11.73 - 52.16)
metastases to skin	4	4.96 (1.86 - 13.24)	metastases to pelvis	7	23.01 (10.92 - 48.5)
tumour rupture	3	7.01 (2.25 -	hepatic cancer	6	13.7 (6.14 -

			21.8)	metastatic		30.59)
	head and neck cancer	3	5.36 (1.72 - 16.68)	tumour haemorrhage	6	4.33 (1.94 - 9.65)
	hepatic cancer metastatic	3	4.07 (1.31 - 12.66)	pyogenic granuloma	6	25.79 (11.52 - 57.74)
	rectal cancer metastatic	3	10.53 (3.38 - 32.84)	tumour necrosis	5	9.45 (3.92 - 22.76)
	metastases to chest wall	3	10.85 (3.48 - 33.85)	tumour perforation	5	35.34 (14.58 - 85.64)
	/	/	/	metastases to bladder	4	31.37 (11.67 - 84.3)
	/	/	/	haemangioma	4	3.72 (1.39 - 9.92)
	/	/	/	lymphangiosis carcinomatosa	4	7.47 (2.8 - 19.95)
	/	/	/	rectosigmoid cancer metastatic	4	297.99 (103.39 - 858.88)
	/	/	/	pancreatic carcinoma metastatic	4	2.72 (1.02 - 7.25)
	/	/	/	tumour pain	4	5.93 (2.22 - 15.83)
	/	/	/	head and neck cancer	4	12 (4.49 - 32.08)
	/	/	/	colon cancer stage iv	3	9.91 (3.19 - 30.84)
	/	/	/	colorectal adenocarcinoma	3	30.47 (9.73 - 95.41)
**NERVOUS SYSTEM DISORDERS**	loss of consciousness	222	2.07 (1.82 - 2.37)	neuropathy peripheral	316	7.1 (6.35 - 7.93)
	syncope	153	1.82 (1.55 - 2.13)	peripheral sensory neuropathy	96	34.83 (28.45 - 42.64)
	neuropathy peripheral	147	1.95 (1.66 - 2.29)	polyneuropathy	81	14.2 (11.41 - 17.68)
	speech disorder	113	2.54 (2.11 - 3.06)	neurotoxicity	66	8.28 (6.5 - 10.55)
	unresponsive to stimuli	72	3.44 (2.73 - 4.33)	dysgeusia	54	1.4 (1.07 - 1.83)
	coma	57	1.44 (1.11 - 1.86)	cerebral infarction	23	1.86 (1.23 - 2.8)
	cerebral infarction	47	2.27 (1.7 - 3.02)	peripheral motor neuropathy	5	7.22 (3 - 17.38)
	cerebral ischaemia	33	7.26 (5.16 - 10.23)	acute polyneuropathy	3	25.79 (8.25 - 80.61)
	neurotoxicity	30	2.24 (1.56 -	cholinergic syndrome	3	9.82 (3.16 -

			3.2)			30.55)
	polyneuropathy	25	2.6 (1.75 - 3.85)	/	/	/
	peripheral sensory neuropathy	18	3.83 (2.41 - 6.08)	/	/	/
	peripheral motor neuropathy	14	12.16 (7.18 - 20.59)	/	/	/
	vocal cord paralysis	5	3.34 (1.39 - 8.04)	/	/	/
	consciousness fluctuating	4	4.8 (1.8 - 12.81)	/	/	/
	cerebellar infarction	4	2.77 (1.04 - 7.4)	/	/	/
	autonomic neuropathy	3	3.69 (1.19 - 11.47)	/	/	/
**PSYCHIATRIC DISORDERS**	mental status changes	58	2.51 (1.94 - 3.25)	/	/	/
	delirium	43	1.54 (1.14 - 2.07)	/	/	/
	eating disorder	36	1.97 (1.42 - 2.73)	/	/	/
	staring	5	2.46 (1.02 - 5.92)	/	/	/
**PRODUCT ISSUES**	/	/	/	product temperature excursion issue	5	3.17 (1.32 - 7.63)
**RENAL AND URINARY DISORDERS**	urinary incontinence	38	1.52 (1.1 - 2.08)	proteinuria	27	2.96 (2.03 - 4.31)
	hydronephrosis	18	2.9 (1.82 - 4.6)	hydronephrosis	19	5.13 (3.27 - 8.04)
	ureteric obstruction	8	6.93 (3.46 - 13.89)	nephrotic syndrome	9	2.7 (1.4 - 5.19)
	renal tubular disorder	8	3.29 (1.64 - 6.58)	renal tubular disorder	5	3.44 (1.43 - 8.27)
	prerenal failure	6	5.14 (2.3 - 11.46)	iga nephropathy	5	7.49 (3.11 - 18.02)
	urinary tract obstruction	6	3.01 (1.35 - 6.71)	ureteric obstruction	4	5.79 (2.17 - 15.44)
	glomerulonephritis rapidly progressive	4	4.61 (1.73 - 12.31)	urinoma	3	41.26 (13.13 - 129.61)
	ureterolithiasis	3	3.59 (1.16 - 11.16)	prerenal failure	3	4.29 (1.38 - 13.33)
	hydroureter	3	10.43 (3.34 - 32.52)	/	/	/

**REPRODUCTIVE SYSTEM AND BREAST DISORDERS**	genital erythema	3	8.25 (2.65 - 25.7)	genital rash	4	5.6 (2.1 - 14.95)
	/	/	/	female genital tract fistula	4	4.55 (1.7 - 12.13)
	/	/	/	genital ulceration	3	9.26 (2.98 - 28.81)
**RESPIRATORY, THORACIC AND MEDIASTINAL DISORDERS**	dyspnoea	957	2.05 (1.92 - 2.19)	interstitial lung disease	239	10.33 (9.09 - 11.74)
	pulmonary embolism	303	3.75 (3.35 - 4.2)	pulmonary embolism	141	2.91 (2.47 - 3.44)
	interstitial lung disease	220	5.65 (4.95 - 6.46)	pneumonitis	43	3.38 (2.51 - 4.56)
	respiratory failure	196	3.23 (2.81 - 3.72)	lung disorder	34	1.42 (1.01 - 1.98)
	pneumonitis	133	6.28 (5.29 - 7.45)	hypoxia	31	1.81 (1.27 - 2.57)
	pleural effusion	125	2.43 (2.04 - 2.9)	respiratory distress	27	1.97 (1.35 - 2.87)
	hypoxia	117	4.09 (3.41 - 4.91)	bronchospasm	20	2.77 (1.79 - 4.3)
	epistaxis	97	1.54 (1.26 - 1.87)	acute respiratory distress syndrome	17	1.94 (1.2 - 3.12)
	bronchospasm	96	8.01 (6.55 - 9.79)	pulmonary toxicity	16	4.92 (3.01 - 8.04)
	respiratory arrest	85	3.52 (2.84 - 4.35)	pulmonary artery thrombosis	12	31.47 (17.78 - 55.69)
	respiratory distress	82	3.58 (2.88 - 4.44)	hiccups	10	2.54 (1.37 - 4.73)
	pharyngeal inflammation	77	83.46 (66.18 - 105.27)	pneumomediastinum	9	11.08 (5.75 - 21.33)
	wheezing	73	1.55 (1.23 - 1.95)	laryngospasm	5	3.67 (1.52 - 8.81)
	acute respiratory distress syndrome	69	4.71 (3.72 - 5.97)	oropharyngeal blistering	5	5.87 (2.44 - 14.11)
	haemoptysis	60	2.54 (1.97 - 3.27)	pulmonary infarction	4	3.19 (1.2 - 8.52)
	pneumothorax	54	4.11 (3.15 - 5.37)	pulmonary pain	4	2.85 (1.07 - 7.59)
	aspiration	50	5.71 (4.32 - 7.54)	malignant pleural effusion	4	4.72 (1.77 - 12.59)
	tachypnoea	47	4.3 (3.23 - 5.72)	pulmonary granuloma	3	5.16 (1.66 - 16.03)
	throat tightness	45	2.01 (1.5 -	nasal inflammation	3	6.08 (1.96 -

		2.7)			18.89)
laryngeal oedema	37	7.22 (5.23 - 9.98)	nasal ulcer	3	3.63 (1.17 - 11.27)
lung infiltration	35	4.43 (3.18 - 6.17)	pharyngeal ulceration	3	4.51 (1.45 - 14.02)
pulmonary fibrosis	24	1.56 (1.04 - 2.33)	/	/	/
atelectasis	24	2.85 (1.91 - 4.26)	/	/	/
pharyngeal oedema	23	1.62 (1.08 - 2.44)	/	/	/
obstructive airways disorder	22	2.28 (1.5 - 3.47)	/	/	/
asphyxia	21	3.07 (2 - 4.71)	/	/	/
pulmonary haemorrhage	17	2.51 (1.56 - 4.05)	/	/	/
hiccups	17	2.58 (1.6 - 4.16)	/	/	/
apnoea	15	2.25 (1.35 - 3.73)	/	/	/
stridor	14	5.73 (3.39 - 9.7)	/	/	/
pneumomediastinum	12	8.83 (5 - 15.59)	/	/	/
laryngeal disorder	9	14.96 (7.75 - 28.89)	/	/	/
increased bronchial secretion	7	3.84 (1.83 - 8.07)	/	/	/
pharyngeal haemorrhage	7	6.69 (3.18 - 14.06)	/	/	/
lung consolidation	7	3.02 (1.44 - 6.33)	/	/	/
bronchial obstruction	7	5.69 (2.71 - 11.95)	/	/	/
pleuritic pain	7	2.91 (1.39 - 6.12)	/	/	/
tracheal stenosis	6	15.32 (6.84 - 34.3)	/	/	/
diffuse alveolar damage	6	6.07 (2.72 - 13.54)	/	/	/
pulmonary artery thrombosis	6	9.31 (4.17 - 20.79)	/	/	/

	nasal ulcer	5	3.62 (1.5 - 8.71)	/	/	/
	alveolitis	4	3.17 (1.19 - 8.45)	/	/	/
	malignant pleural effusion	4	2.82 (1.06 - 7.51)	/	/	/
	rhonchi	4	3.31 (1.24 - 8.83)	/	/	/
	pharyngeal paraesthesia	4	3.35 (1.25 - 8.94)	/	/	/
	pharyngeal ulceration	4	3.6 (1.35 - 9.6)	/	/	/
	laryngeal necrosis	3	84.25 (26.01 - 272.96)	/	/	/
	respiratory tract oedema	3	5.41 (1.74 - 16.82)	/	/	/
	laryngeal discomfort	3	5.96 (1.92 - 18.55)	/	/	/
	bronchostenosis	3	6.63 (2.13 - 20.63)	/	/	/
	pharyngeal stenosis	3	34.43 (10.9 - 108.7)	/	/	/
	sputum retention	3	4.05 (1.3 - 12.58)	/	/	/
	lung cyst	3	9.28 (2.98 - 28.92)	/	/	/
	pulmonary cavitation	3	4.37 (1.41 - 13.59)	/	/	/
**SKIN AND SUBCUTANEOUS TISSUE DISORDERS**	rash	1619	4.76 (4.53 - 5)	rash	1204	5.97 (5.63 - 6.32)
	dermatitis acneiform	574	132.56 (121.49 - 144.62)	dermatitis acneiform	651	258.74 (238.16 - 281.1)
	pruritus	509	1.76 (1.61 - 1.92)	skin toxicity	437	198.52 (179.73 - 219.27)
	erythema	389	2.28 (2.07 - 2.52)	dry skin	353	5.79 (5.21 - 6.43)
	dry skin	313	3.05 (2.73 - 3.41)	pruritus	249	1.44 (1.27 - 1.63)
	acne	251	3.84 (3.39 - 4.35)	skin disorder	230	14.16 (12.43 - 16.13)
	urticaria	241	1.81 (1.59 - 2.05)	erythema	215	2.11 (1.85 - 2.42)
	skin reaction	178	15.87 (13.69 - 18.41)	skin reaction	185	27.73 (23.97 - 32.07)

hyperhidrosis	163	1.49 (1.28 - 1.74)	acne	175	4.48 (3.86 - 5.2)
skin disorder	144	5.25 (4.46 - 6.19)	skin fissures	136	17.32 (14.63 - 20.52)
skin toxicity	139	34.66 (29.27 - 41.05)	dermatitis	95	10.17 (8.31 - 12.44)
skin fissures	136	10.32 (8.72 - 12.22)	palmar-plantar erythrodysaesthesia syndrome	93	7.97 (6.5 - 9.77)
dermatitis	121	7.74 (6.47 - 9.25)	skin exfoliation	92	2.34 (1.9 - 2.87)
skin exfoliation	119	1.8 (1.51 - 2.16)	skin lesion	66	4.86 (3.82 - 6.19)
rash pruritic	90	2.08 (1.69 - 2.55)	rash maculo-papular	58	5.45 (4.21 - 7.05)
nail disorder	89	13.42 (10.89 - 16.55)	rash pruritic	56	2.16 (1.66 - 2.81)
rash erythematous	72	2.03 (1.61 - 2.56)	skin ulcer	47	3.62 (2.72 - 4.82)
palmar-plantar erythrodysaesthesia syndrome	67	3.42 (2.69 - 4.34)	pain of skin	44	3.19 (2.38 - 4.29)
skin ulcer	49	2.25 (1.7 - 2.98)	rash erythematous	44	2.08 (1.55 - 2.79)
skin lesion	44	1.93 (1.44 - 2.59)	skin haemorrhage	33	5.15 (3.66 - 7.25)
skin haemorrhage	39	3.64 (2.65 - 4.98)	scab	32	5.79 (4.09 - 8.19)
rash maculo-papular	39	2.18 (1.59 - 2.99)	rash papular	31	2.7 (1.9 - 3.84)
rash papular	38	1.98 (1.44 - 2.72)	nail disorder	28	7.01 (4.83 - 10.16)
onychoclasis	33	5.8 (4.12 - 8.16)	photosensitivity reaction	28	3.38 (2.33 - 4.89)
pain of skin	33	1.43 (1.02 - 2.01)	pigmentation disorder	23	7.51 (4.98 - 11.31)
scab	33	3.56 (2.53 - 5.02)	stevens-johnson syndrome	22	1.96 (1.29 - 2.98)
hair growth abnormal	30	5.17 (3.61 - 7.4)	purpura	21	4.71 (3.07 - 7.23)
cold sweat	28	1.9 (1.31 - 2.76)	drug eruption	20	2.37 (1.53 - 3.67)
dermatitis allergic	21	2.16 (1.41 - 3.31)	hypertrichosis	18	18.47 (11.61 - 29.39)

purpura	20	2.68 (1.72 - 4.15)	papule	16	4.86 (2.97 - 7.94)
nail bed inflammation	16	65.95 (39.8 - 109.27)	toxic skin eruption	15	3.02 (1.82 - 5.01)
skin necrosis	16	3.61 (2.21 - 5.9)	xeroderma	14	121.55 (70.73 - 208.88)
cutaneous symptom	15	29.38 (17.59 - 49.08)	onychoclasis	13	3.81 (2.21 - 6.57)
exfoliative rash	14	6.52 (3.86 - 11.03)	erythema multiforme	13	2.8 (1.62 - 4.82)
dermatitis exfoliative	14	3.13 (1.85 - 5.29)	nail bed inflammation	13	88.74 (50.84 - 154.9)
papule	14	2.54 (1.5 - 4.29)	skin hyperpigmentation	12	2.8 (1.59 - 4.94)
hypertrichosis	13	7.94 (4.6 - 13.7)	sensitive skin	12	3.81 (2.16 - 6.72)
onychomadesis	13	4.49 (2.61 - 7.75)	hair growth abnormal	11	3.16 (1.75 - 5.72)
nail discolouration	12	3.63 (2.06 - 6.39)	skin necrosis	10	3.77 (2.03 - 7.02)
onychalgia	10	12.25 (6.57 - 22.86)	exfoliative rash	10	7.79 (4.18 - 14.5)
onycholysis	8	6.35 (3.17 - 12.73)	onycholysis	9	11.98 (6.22 - 23.08)
rash vesicular	8	2.14 (1.07 - 4.29)	hyperkeratosis	9	3.24 (1.68 - 6.22)
ingrowing nail	7	3.42 (1.63 - 7.18)	onychomadesis	8	4.62 (2.31 - 9.26)
rash follicular	7	56.6 (26.46 - 121.06)	rash vesicular	7	3.14 (1.5 - 6.59)
nail bed bleeding	7	15.66 (7.43 - 33.03)	nail discolouration	7	3.54 (1.69 - 7.43)
livedo reticularis	6	3.5 (1.57 - 7.8)	skin erosion	7	4.69 (2.23 - 9.84)
seborrhoeic dermatitis	6	4.62 (2.07 - 10.3)	seborrhoeic dermatitis	6	7.73 (3.47 - 17.25)
palmar erythema	5	5.22 (2.17 - 12.57)	ingrowing nail	5	4.09 (1.7 - 9.83)
nail bed disorder	4	11.57 (4.32 - 30.99)	hirsutism	5	7.31 (3.04 - 17.59)
skin oedema	4	4.7 (1.76 - 12.54)	mucocutaneous rash	5	77.07 (31.48 - 188.67)
subcutaneous emphysema	4	5.13 (1.92 - 13.7)	cutaneous symptom	5	16.11 (6.68 - 38.86)

	hirsutism	4	3.49 (1.31 - 9.31)	livedo reticularis	5	4.89 (2.03 - 11.75)
	nail toxicity	4	18.64 (6.94 - 50.1)	onychalgia	5	10.21 (4.24 - 24.58)
	hypotrichosis	3	5.1 (1.64 - 15.85)	henoch-schonlein purpura	4	3.96 (1.49 - 10.57)
	nail bed tenderness	3	31.08 (9.86 - 97.97)	panniculitis	4	3.3 (1.24 - 8.79)
	/	/	/	hypersensitivity vasculitis	4	2.8 (1.05 - 7.46)
	/	/	/	rash papulosquamous	3	38.04 (12.12 - 119.37)
	/	/	/	dermatosis	3	9.91 (3.19 - 30.84)
	/	/	/	perioral dermatitis	3	12.71 (4.08 - 39.57)
	/	/	/	skin oedema	3	5.89 (1.9 - 18.31)
	/	/	/	rash follicular	3	39.44 (12.56 - 123.82)
	/	/	/	intertrigo	3	11.79 (3.79 - 36.69)
**SOCIAL CIRCUMSTANCES**	refusal of treatment by patient	11	1.93 (1.07 - 3.48)	refusal of treatment by patient	14	4.11 (2.43 - 6.95)
	social problem	7	2.14 (1.02 - 4.5)	/	/	/
**SURGICAL AND MEDICAL PROCEDURES**	tracheostomy	13	11.27 (6.53 - 19.47)	surgery	52	1.99 (1.51 - 2.61)
	hepatectomy	11	50.83 (27.76 - 93.07)	hepatectomy	16	126.63 (76.25 - 210.28)
	cancer surgery	9	11.71 (6.07 - 22.6)	hospice care	10	2.19 (1.18 - 4.07)
	lymphadenectomy	8	9.87 (4.92 - 19.8)	cancer surgery	7	15.23 (7.24 - 32.04)
	leg amputation	8	2.25 (1.12 - 4.5)	colostomy	6	6.34 (2.84 - 14.13)
	gastrostomy	7	5.89 (2.8 - 12.38)	radiotherapy	5	5.93 (2.47 - 14.27)
	ileostomy	6	3.12 (1.4 - 6.94)	liver operation	5	38.53 (15.89 - 93.46)
	gastrointestinal tube insertion	6	3.02 (1.36 - 6.74)	depilation	5	372.49 (142.11 - 976.35)
	resuscitation	6	4.19 (1.88 - 9.33)	nephrostomy	3	20.16 (6.46 - 62.92)

	colostomy	5	3.15 (1.31 - 7.58)	palliative care	3	4.5 (1.45 - 13.98)
	tumour excision	4	6.1 (2.28 - 16.3)	/	/	/
	gastrointestinal surgery	4	6.56 (2.45 - 17.53)	/	/	/
	proctectomy	4	20.23 (7.52 - 54.41)	/	/	/
	liver operation	3	13.68 (4.38 - 42.73)	/	/	/
	laryngectomy	3	55.2 (17.3 - 176.18)	/	/	/
	colon operation	3	3.26 (1.05 - 10.14)	/	/	/
	colostomy closure	3	11.81 (3.79 - 36.86)	/	/	/
**VASCULAR DISORDERS**	hypotension	660	4 (3.71 - 4.32)	deep vein thrombosis	71	2.11 (1.67 - 2.67)
	flushing	235	2.71 (2.39 - 3.08)	embolism	21	4.93 (3.21 - 7.57)
	deep vein thrombosis	198	3.53 (3.07 - 4.06)	venous thrombosis	16	8.16 (4.99 - 13.33)
	thrombosis	95	1.39 (1.14 - 1.71)	infarction	9	2.47 (1.29 - 4.75)
	cyanosis	68	5.14 (4.05 - 6.52)	vena cava thrombosis	8	9.45 (4.72 - 18.93)
	pallor	52	2.24 (1.71 - 2.94)	hypovolaemic shock	7	3.02 (1.44 - 6.35)
	shock	43	2.34 (1.74 - 3.16)	venous thrombosis limb	7	6.15 (2.93 - 12.91)
	circulatory collapse	43	2.93 (2.17 - 3.96)	embolism arterial	6	9.97 (4.47 - 22.24)
	embolism	31	4.35 (3.06 - 6.2)	jugular vein thrombosis	6	6.81 (3.05 - 15.18)
	venous thrombosis	20	6.09 (3.93 - 9.46)	embolism venous	6	4.11 (1.85 - 9.17)
	peripheral ischaemia	19	5.54 (3.53 - 8.69)	thrombophlebitis	6	3.28 (1.47 - 7.3)
	hypovolaemic shock	18	4.65 (2.93 - 7.39)	hyperaemia	4	4.07 (1.53 - 10.87)
	vena cava thrombosis	16	11.34 (6.93 - 18.56)	superior vena cava syndrome	3	9.75 (3.14 - 30.33)
	venous thrombosis limb	13	6.84 (3.96 - 11.79)	/	/	/

jugular vein thrombosis	13	8.84 (5.12 - 15.27)	/	/	/
subclavian vein thrombosis	12	12.16 (6.89 - 21.49)	/	/	/
hyperaemia	12	7.33 (4.16 - 12.94)	/	/	/
arterial haemorrhage	9	9.77 (5.07 - 18.84)	/	/	/
phlebitis	9	2.02 (1.05 - 3.89)	/	/	/
peripheral arterial occlusive disease	8	2.32 (1.16 - 4.65)	/	/	/
axillary vein thrombosis	7	23.35 (11.04 - 49.37)	/	/	/
aortic thrombosis	6	6.64 (2.97 - 14.81)	/	/	/
pelvic venous thrombosis	6	4.86 (2.18 - 10.83)	/	/	/
thrombophlebitis migrans	6	26.91 (11.97 - 60.5)	/	/	/
peripheral artery thrombosis	5	4.29 (1.78 - 10.32)	/	/	/
arterial thrombosis	5	3.04 (1.26 - 7.31)	/	/	/
extremity necrosis	5	3.43 (1.43 - 8.26)	/	/	/
embolism arterial	4	3.96 (1.48 - 10.57)	/	/	/
hypoperfusion	3	3.55 (1.14 - 11.04)	/	/	/
superficial vein thrombosis	3	6.39 (2.05 - 19.88)	/	/	/
femoral artery embolism	3	57.17 (17.9 - 182.66)	/	/	/

Abbreviations: FAERS, FDA Adverse Event Reporting System; PTs, preferred terms; ROR, reported odds ratio; SOCs, systemic organ classes.

Supplementary Table 6. The top 20 concomitant drugs with cetuximab and panitumumab (N=102,125).

**Concomitant drugs**	**Number**	**Ratio (%)**
Fluorouracil	10288	10.07%
Calcium folinate	5837	5.72%
Irinotecan	5081	4.98%
Oxaliplatin	4675	4.58%
Cisplatin	2329	2.28%
Carboplatin	1529	1.50%
Dexamethasone	1411	1.38%
Benadryl	1406	1.38%
Paclitaxel	1237	1.21%
Ondansetron	820	0.80%
Capecitabine	714	0.70%
Bevacizumab	571	0.56%
Docetaxel	448	0.44%
Aspirin	415	0.41%
Polaramine	394	0.39%
Gemcitabine	376	0.37%
Palonosetron	355	0.35%
Prochlorperazine	302	0.30%
Granisetron	299	0.29%
Oxycontin	267	0.26%
